# A review on in-silico analysis of immune cell trafficking and interactions with the tumour microenvironment

**DOI:** 10.3389/fonc.2026.1835062

**Published:** 2026-07-03

**Authors:** Kharan P., Amy S. Mathew, Payel Ghosh, Syama H. P.

**Affiliations:** 1 Department of Biomedical Sciences, School of Bioscience and Technology, Vellore Institute of Technology, Vellore, Tamil Nadu, India; 2 Laboratory of Immunopharmacology and Experimental Therapeutics, Division of Cancer Research, Regional Cancer Centre (RCC), Thiruvananthapuram, Kerala, India; 3 Department of Integrative Biology, School of Bioscience and Technology, Vellore Institute of Technology, Vellore, Tamil Nadu, India

**Keywords:** digital twin technology, immunotherapy, multi omics, transcriptomics, tumor microenvironment (TME)

## Abstract

The tumour microenvironment (TME) contains a diverse mix of cells and components, including cancer cells, immune cells, connective tissue, and biochemical factors; all of these are constantly interacting as well as influencing both the progression and spread of cancer and the ability of the immune system to recognise and respond to it. Accumulating evidence indicates that immune cell trafficking in TMEs is a major factor in determining whether tumours are destroyed by immune defences or evade immune surveillance. However, advances in experimental techniques do not provide a complete picture of how immune-tumour cell interactions occur with respect to their spatial, temporal, and molecular characteristics. This review examines existing in silico tools for evaluating how immune cells migrate, communicate, and function in the TME. The components that affect how immune cells infiltrate tumours will be summarized (i.e., chemotactic gradients, adhesion molecules, extracellular matrix remodelling, hypoxia, and metabolic reprogramming), and their role in immune exclusion and the development of immune escape will be emphasized. Computational modelling techniques (e.g., agent-based models, ordinary and partial differential equation models, systems biology models, network biology models, and machine learning prediction models) enable multiscale simulation of immune dynamics. This capability helps further our understanding of how tumours escape immune surveillance and develop into malignancies. The use of bioinformatics databases and major bioinformatics resources such as TCGA, TIMER, TCIA, etc., that may assist in understanding the composition of the immune system and immunogenomics of tumours is assessed. To demonstrate the predictive ability of computational models to establish patterns of immune cell traffic and predict the efficacy of immunotherapy, we examine specific in silico analyses of distinct immune cell populations, such as Tumour-associated macrophages (TAMs) and myeloid-derived suppressor cells (MDSCs). Furthermore, integrating multi-omics and spatial transcriptomic datasets enables personalised modelling of potential responses to immune checkpoint therapy. Despite these advantages, precision immunotherapy faces many challenges, including data heterogeneity, model validation, and translation limitations, as well as future perspectives on precision immunotherapy using digital twin technology. Overall, the findings from this review support the increasing relevance of bioinformatics and computational science in understanding immune-TME interactions and developing novel cancer immunotherapies.

## 1 Introduction

In the past, cancer was viewed solely as an abnormal expression of genes at both the molecular and cellular levels. The interrelationship between tumour cells and the TME has been identified as one of the most critical factors influencing tumour initiation, progression, and resistance to treatment (1). The complex ecosystem comprises diverse immune cells, stromal cells, cancer-associated fibroblasts, and a dense network of tumour vasculature, all of which drive cancer initiation (1). The cellular composition and infiltration patterns within this architecture determine clinical outcomes, while certain stromal elements facilitate lymphatic dissemination and support cancer cell survival. Robust infiltration of effector immune cells directly correlates with prolonged survival and enhances therapeutic efficacy in colorectal, breast, and liver cancers (2).

The level and type of immune cells in the TME are fundamental determinants of treatment outcomes and treatment progress; rising levels of effector (CD^8+^) T cells have been consistently linked to a more favourable clinical prognosis in patients with melanoma, colorectal, and breast cancers (3). Increased levels of Tregs and TAMs have been associated with poorer clinical outcomes and immune escape in the above-stated cancers (2). This cellular composition is not static but is shaped by vigorous crosstalk between the tumour and the network of components surrounding it. The crosstalk between the tumour and the TME is dynamic, affecting tumour growth (4). For instance, CAFs, ECM, and vesicles remodel the TME, promoting tumour growth and establishing an immunosuppressive TME that compromises the efficacy of immunotherapies (5). It is challenging to experimentally characterize the diverse and ever-changing types of interactions with this type of material because of their dynamic and heterogeneous nature; consequently, there is a growing demand for alternative computational/in silico modelling methodologies (1). Treatment response may not be consistent; therefore, the role of computational tools in understanding the molecular-level dynamics between immune cells and tumour is of great importance (6).

Advanced computational models and bioinformatic tools now allow analysis of the TME at the transcriptomic, proteomic, and spatial levels (7). Popular tools include CIBERSORT, which utilizes RNA-based assessments of immune cell populations within a given sample. It can be a broadly deployed tool for performing immune deconvolution analyses; its utility is primarily reliant on the quality of the input bulk RNA-seq data and the accuracy with which the immune signature has been predefined. Therefore, in TME environments that are highly heterogeneous, using bulk transcriptomic methods becomes increasingly problematic because of their inability to accurately resolve rare immune cell subpopulations as well as spatially discrete cellular interactions, and ESTIMATE, which allows for quantification of tumour purity along with stromal and immune scores, gives a fast insight into the patterns of infiltrative and immune infiltration; however, it doesn’t have a single cell resolution or record the different types of cells in different spaces within the environment of a tumour. (8, 9/re-validated by 10). These tools allow researchers to assess immune cell infiltration patterns by analysing TCGA data; these sets are essential for identifying immune subtypes with prognostic algorithm potential. In recent large-scale immunogenomics studies, the utility of bulk transcriptomic data has been demonstrated. The increasing heterogeneity of tumours and the dynamic nature of immune cell movement have highlighted some of the limitations of using bulk transcriptomic data (11). Therefore, recent studies favour the use of single-cell RNA sequencing or spatial transcriptomics to provide a more detailed analysis of the location and functional state of immune cells in the tumour environment. (12). Increased graph-based computational frameworks and the use of Graph Neural Networks (GNNs) have also enhanced the ability to model spatially defined immune cell interactions and communication networks within tumour tissue. The validation of simulated immune interactions remains a significant barrier to computational oncology, particularly in transitioning simulation-based findings into patient-specific therapies.

Single-cell RNA sequencing (scRNA-Seq) allows measurement of gene expression in each individual cell, providing detailed and fine-grained information, but it is expensive, whereas bulk RNA-seq deconvolution cannot provide the same comprehensive information as single-cell experiments, but it offers a more affordable way to extract cell type information from existing bulk datasets (13). However, deconvolution accuracy varies across datasets due to differences in data processing, normalization, and the specific preservation methods used and also with dampened weighted least squares outperforming others in most but not all cases, and reference selection either using scRNA-seq vs bulk-derived profiles, which strongly impacts performance (14).

TMEs comprise diverse cell populations, including immune cells and the extracellular matrix (ECM) (15). All work together to create an environment that enables tumour evolution; as a result, such an environment is referred to as a pro-TME (16). Immune cells must invade and traverse the TME before they can effectively fight the tumour (17). The ability of key immune cell populations, including CD8^+^ cells, NK cells, and dendritic cells, to move from one place to another depends on their ability to migrate through chemokine gradients and adhere to surfaces via adhesion molecules (18). When the immune system fails to regulate suppressive immune populations, including Tregs, MDSCs, and TAMS, they accumulate within the TME (19). All currently available experimental techniques, which offer only partial insight into TME dynamics and tumour development, are limited by ethical issues, high costs, and the inability to capture dynamic changes in the TME over time (20). Alternatively, in silico (agent-based simulations, ODEs/PDEs, etc.) methods provide researchers with a means by which to simulate cellular interactions within the TME without the above-mentioned limitations (6).

The principal aim of this review is to describe the use of bioinformatics-based in silico analyses of immune cell trafficking, along with their interactions within the TME, can be used to investigate the complex and evolving interactions between immune responses and the TME (21). This review also examines the role of key components and variable behaviours within TME, migratory and communicative patterns of cells of the immune system to the neighbouring TME structures (22), individual in silico methodologies/approaches for each type of immune cells (e.g. T-cells; NK-cells; dendritic; TAM; MDSC), and the creation of immune checkpoint models (e.g., PD-1/PD-L1; CTLA-4; and new antibody therapies) (23).

The combination of multi-layered omics data encompassing genomic, transcriptomic, proteomic, and metabolomics profiles, alongside single- cell and spatial transcriptomics, and the clinical relevance of using immune checkpoint therapies (e.g., predicting individual therapy responses; developing personalised models to assess individual therapy response; and modelling drug/TME interactions) (24). Current obstacles related to the quality/validation/resolution of data in this field, and future goals to improve scientific understanding of molecular mechanisms responsible for tumour (neoplastic) development, progression, and response to therapies (25).

## 2 Tumour microenvironment: components and dynamics

**Figure 1:**
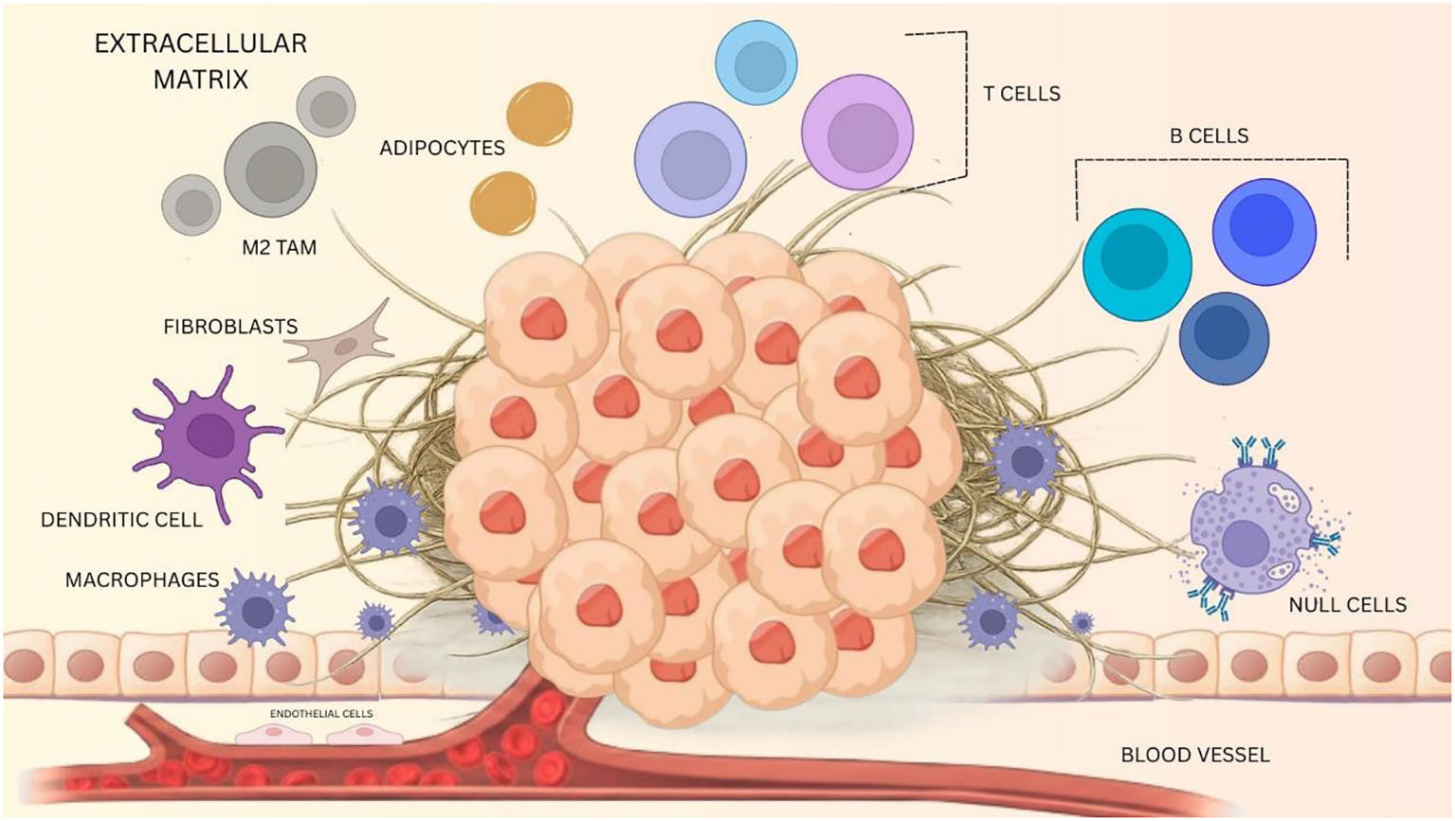
Cellular heterogeneity in tumour microenvironment.

A TME, with both malignant and non-malignant cells, is an ever-evolving, diverse ecosystem that controls processes involved in tumour growth, including tumour development, expansion, invasion, metastasis, immune evasion, and resistance to treatment (26). The TME comprises cellular and non-cellular components, all of which interact through soluble factors, extracellular matrix remodelling, and biomechanical forces, which together influence the rate of tumour progression (27). Combined with the above factors, these successive interactions also govern how immune cells are trafficked, enter the tumour, and functionally polarise in the respective tissues in which they reside (28). **Figure 1** visualises the cellular heterogeneity in tumour microenvironment.

### 2.1 Cellular components (immune, stromal, endothelial)

The immune components of the TME, which induce an anti-cancer response in the immune system (28). Immunologically inhibitory cell types include Tregs, TAMs, and MDSCs, which create an environment of immune tolerance and decrease effector cell activity (29). The ECM is remodelled by CAFs, which secrete growth factors that build both physical and chemical barriers that block immune cells from migrating into tumours (30). Tumours have an abnormal architecture (31) and an overall reduction in adhesion molecule expression on the endothelial lining of tumour-associated blood vessels, leading to impaired migration of leukocytes out of the blood and into the tumour core.

### 2.2 Non-cellular components (ECM, cytokines, chemokines)

The ECM provides structural support for other parts of the TME, such as stromal cells (32), and can also support the function of its cellular components. In addition to providing a framework for other elements of the TME, the properties of the ECM (such as stiffness, how ECM is reconstructed by matrix metalloproteinases, and how it is cross- linked) will influence the movement of immune cells within the TME, as well as their function and location (33). Immunosuppressive cytokines such as IL-10 and TGF-β establish an immunosuppressive gradient within the TME, which both limit the function of immune effectors and provide a spatial niche for recruiting immunosuppressive cells from the “inflammatory” component (29). Gradients of specific chemokines (e.g., CXCL12, CCL2, CXCL9, and CXCL10) generate an attractive environment for MDSCs in the hypoxic regions of tumours, as well as an inhibitory environment for effector T cells in those regions (34). Modelling both the diffusion and receptor signals produced by chemokines using partial differential equations indicates that changes in trafficking patterns driven by these factors will differ depending on the conditions in the TME at the time (35). In addition, the ECM stiffness may provide signals that drive macrophage polarization into a pro-tumourogenic phenotype (33). Signalling factors, such as tumour- and stroma-derived chemokines and cytokines, bidirectionally signal to one another through various pathways (27).

Activation of hypoxia-inducible factor (HIF) by low oxygen levels leads to increased secretion of growth factors and immunosuppressive cytokines (36). Consequently, the TME becomes occupied by non-cellular elements, further contributing to the immunosuppressive environment. The non-cellular components of TME include secreted exosomes, which transmit bioactive molecules via horizontal transfer (37), and metabolites found within the ECM (e.g., lactate) that induce M2 macrophage polarization (38). The anti-inflammatory cytokines released by M2 macrophages during inflammation stimulate fibroblast mitosis, leading to the secretion of factors that support angiogenesis and ECM formation. In addition, computational metabolic models are increasingly used to predict Lactate-Mediated Immune Suppression and metabolic exhaustion of Cytotoxic T-Cells within the TME. (39). Metabolic byproducts like lactate, adenosine, kynurenine, and cholesterol are synthesized in the TME and act as dynamic regulators that directly in the TME and act as dynamic regulators that directly influence T cell activity, leading to impacts ranging from reduced proliferation and cytokine production to increased exhaustion and immunosuppression (40). Computational models translate these inputs into cellular states by using kinetic differential equations to compute reaction rates as functions of metabolite and enzyme concentrations, resulting in ODEs that describe metabolite concentration changes over time, enabling the dynamic analysis of biological systems and quantitative prediction of cell states but they are typically limited in scope to one or a few metabolic pathways due to the large number of parameters required, although recent efforts aim to build genome scale kinetic models (41).

### 2.3 Immune evasion pathways in TME

Tumours regulate immune checkpoint pathways to evade detection by the immune system; for instance, the PD-1/PD-L1 axis creates multiple mechanisms of immune evasion, leading to T-cell exhaustion and leading to metabolic changes that deplete effector T cells from functional capacity, e.g., by IDO1-mediated depletion of tryptophan, which can recruit additional suppressor T cells (42). When tumour cells undergo energy metabolic reprogramming via aerobic glycolysis, they deplete essential nutrients and generate immune-inhibitory metabolites, e.g., lactate, thereby impairing the functional capacity of CD8+ T cells (38). These metabolic stresses are aggravated by hypoxic TME, in which HIF-1α mediates dysfunctional angiogenesis, chemokine production, and extracellular matrix rigidity (43), leading to decreased immune cell infiltration and reduced tumour cell detection by the immune system. These overlapping, multi-layered stresses, such as hypoxia or nutrient depletion, are too complex for a traditional laboratory to capture dynamically over time; they can only be understood through mechanistic networks. The integration of virtual networks into mechanistic models enables researchers to understand the dynamics of tumour cells evading immune detection and to predict the restoration of drug-mediated trafficking after the blockade of immune checkpoint pathways or modulation of energy metabolism pathways. However, biological validation of these predicted outcomes remains a significant barrier to success in the theatre of Oncological Biology, due to considerable variability between patients and the dynamic differences in their Tumour Microenvironments. (12).

Ultimately, the effectiveness of these computational frameworks relies on how accurately they capture the molecular pathways for underlying functional exhaustion *in vivo*, at the cellular level, CD^8+^ T cells exhibit a decline in their cytotoxic capabilities when they are induced to another hypoxic stage and experience an excess of PD-L1 ligands (which play a substantial role in inhibiting CD8+ T cell activity) (44). This immune dysfunction is exacerbated by the metabolic byproducts of TME; elevated lactate levels create an acidic environment that inhibits cytotoxicity and NK cell activation (38). Oncometabolites like 2-HG can induce gene expression changes and enable neoplastic cells to evade immune recognition, for instance, by stabilising HIF during hypoxia to modulate the expression of proangiogenic and immunosuppressive genes (45). In addition, hypoxia can facilitate EV release from cells by altering lysosomal homeostasis and activating HIF-1α (46). Collectively, these factors converge on the three primary stressors within TME- hypoxia, acidity, and oxidative stress- which together lead to the impaired cytotoxicity of CD8^+^ T cells. **Table 1** gives an overview of key TME components and their role in immune trafficking and evasion\.

**Table 1:** Overview of key TME components and their role in immune trafficking and evasion.

Component category	Key elements/cell types		Primary functions & roles in the WIS (Wall of Immune Suppression)	Impact on immune trafficking & evasion
Immune Effectors	CD8^+^ T cells, NK cells, Dendritic cells (DCs)		Cytotoxicity, antigen presentation, and molecules support - inflammatory cytokine production.	Facilitate tumour invasion and lysis by actively recruiting immune cells, but are predominantly excluded due to physical and chemical barriers from TME (47).
Suppressive Immune	Tregs, M2-TAMs, MDSCs		Immunosuppressive mediator production (e.g. IL-10, TGF-beta, Lactate).	Provide CCL2 and CCL5 to the TME, reducing the motility and function of effector cells, creating “cold” tumour phenotypes and attracting suppressive cell subsets (48).
Stromal/ECM	CAFs, Collagen, Proteoglycans		Provide structural support, ECM reconstruction, and growth factor production.	Produce physical barriers that are dense and stiff, making it difficult for T- cells to penetrate and providing a microenvironment for M2 polarization (49).
Endothelial /Vascular		Tumour Endothelial Cells (TECs), VEGF	Angiogenesis, regulation of vascular permeability, and nutrient delivery.	Anergic leukocytes due to decreased expression of integrins (e.g. ICAM-1/VCAM-1) on the membrane of tumours prevent leukocytes from being extravasated (47).
Tumour Cells		Malignant cells	Rapidly proliferate, metabolic reprogramming (glycolysis), and release exosomes.	Acidify the TME through Lactate; use of immune checkpoints (PD-L1) and exosomes to alter immune polarization and evade detection (50).
Soluble Mediators		Chemokines (CXCL12, CCL2), DAMPs/LAMPs	Create chemotactic gradients and convert injury to ECM remodelling.	Targeting the blockade of effector cells and the influx of MDSC and T- regs to the TME (51, 52).

## 3 Immune cell trafficking: mechanisms and key regulators

The recruitment and migration of immune cells within the TME are governed by a cascade of events, including rolling, sticking, moving through tissue, and moving in the direction of the chemokines signal (53). Immune cells will arrive in the TME through the coordinated action of chemokines and their receptors, adhesive proteins expressed on the vascular endothelium, and the biophysical characteristics of the TME itself (54). Typically, these factors lead to spatial restriction of functional immune cells within the TME, resulting in negative outcomes for anti-tumour immunity and decreasing the effectiveness of immunotherapies against the tumour (22).

**Figure 2:**
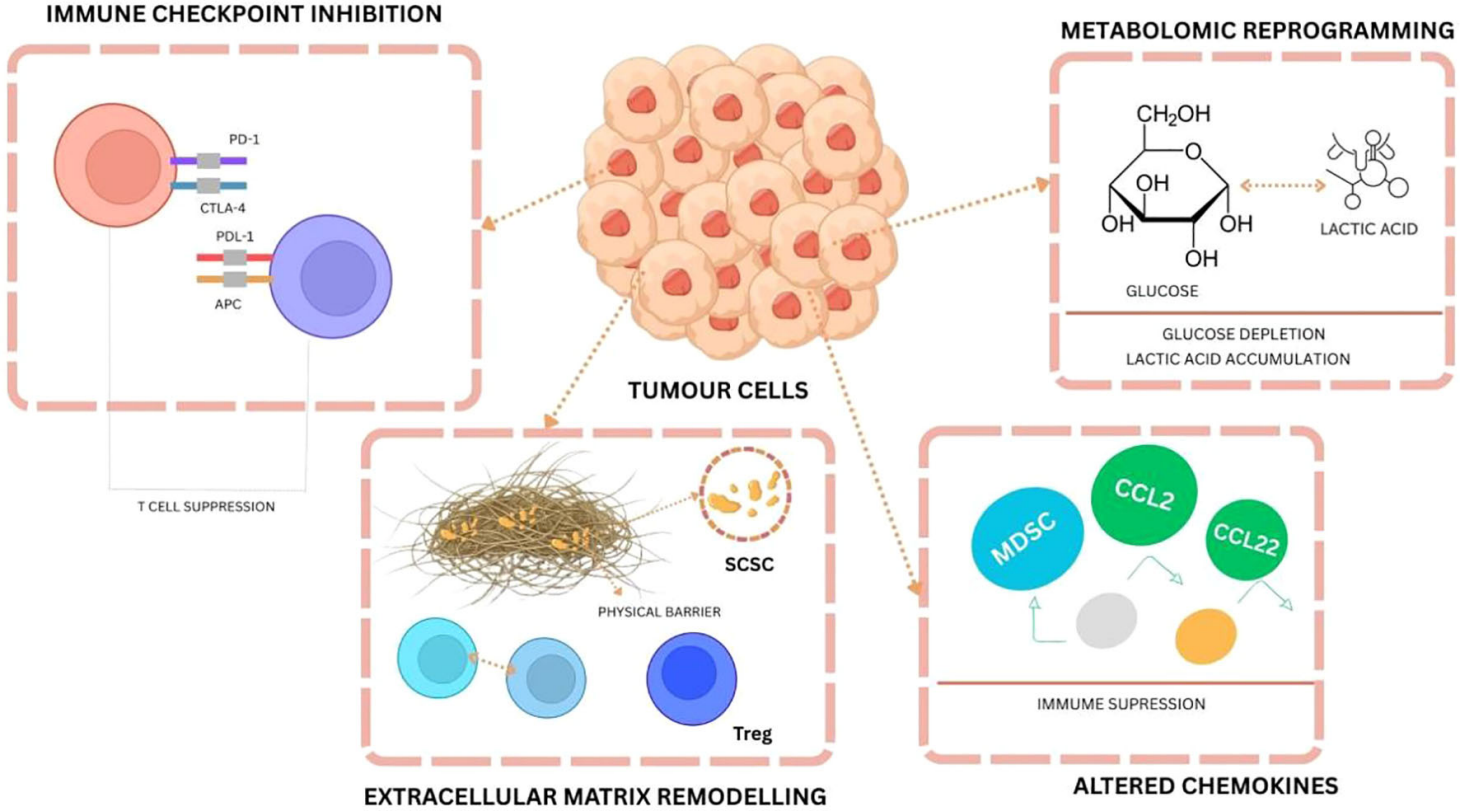
Multimodal Mechanism of Immune Evasion within the TME.

To address these challenges, researchers are increasingly utilizing in silico modelling methodologies (such as Agent-Based Modelling – ABM; Ordinary Differential Equations – ODE; Partial Differential Equations – PDE), and to understand the variables affecting the patterns of immune cell migration and to alter those patterns of immune cell migration by means of chemokines or similar agents, to alter the dynamics of the TME. The accuracy of model predictions depends on parameter selection, the assumptions made, and whether the model has been validated by laboratory experiments. (55). **Figure 2** visualises the Multimodal Mechanism of Immune Evasion within the TME.

### 3.1 Chemokine–chemokine receptor signalling

Immune cells in the TME (56) can be directed to the TME via chemokine gradients. For instance, the CXCL9/10/11-CXCR3 axis recruits NK cells and cytotoxic CD8+ T cells, where they mediate anti-tumour effector function. MDSCs and TAMs are attracted to the TME by the chemokine CCL2, which impairs the capacity of CD8+ T cells to exert their anti-tumour effector function (18). Furthermore, the CXCL12-CXCR4 axis drives MDSC mobilisation and T cells’ exclusion from low-oxygen regions of tumours, thereby supporting immune evasion (57). The disruption of chemokine gradients caused by decoy receptors and sequestration of immune cells from their respective ligands leads to reduced infiltration rates (58).

These dynamics are influenced by the TME itself. For example, hypoxia enhances the expression of CCL2 and CXCL12, thereby promoting the recruitment of suppressive cells and suppressing CXCL9/10, thereby creating a niche that favours the recruitment of immunosuppressive cells (44). Computational modelling has proven essential for understanding these complex interactions. PDE-based models of diffuse chemokine receptors and the chemotactic behaviour of their respective ligands illustrate that the steepness of chemokine gradients significantly affects the ability of immune constituents to migrate towards the TME despite TME heterogeneity (35). By utilising both ABMs and ODEs, the dynamics of receptors, including G-protein activation and Rac/Rho/Cdc42 pathways that govern T cell movement and polarisation, can be modelled together (59). Computational studies have demonstrated that modifying CXCR3 ligands increases CTL trafficking into the TME and enhances CTL responsiveness to anti-PD-1 therapy (60).

### 3.2 Adhesion molecules

The multitude of the features offered by the adhesion molecules perform a fundamental role as part of the multi-step cascade of extravasation; this is accomplished through the selectins initial role in mediation of the rolling interaction, activation of the integrins like VLA-4/VCAM- 1; LFA-1/ICAM-1, by chemokines promotes the firm arrest of the leukocyte from rolling, and trans-migration occurs through the PECAM-1 and JAMs (53). In the TME, however, this cascade is disrupted, where it renders the endothelium incapable of producing ICAM-1 and VCAM-1, which results in poor adhesion and thus poor infiltration of leukocytes (61). Furthermore, endothelial cells in a solid tumour express the αvβ3 and αvβ5 integrins, which promote abnormal angiogenesis and impede the ability of leukocytes to enter the tumour (62). Computer model simulations of adhesion dynamics have shown that integrin activation kinetics can significantly affect the rolling-to-arrest transition of a leukocyte to an endothelial cell under shear flow conditions (35). Agent- Based Models consider both the strength of adhesion and the role of chemokine co-signalling to assess the mechanical barriers created by TME stiffness to infiltration (55). ICAM-1 upregulation induced by cytokines enhances CD8+ T cell infiltration into inflamed tumours (63).

### 3.3 Migration and infiltration patterns

Two different methods of migration are classified as amoeboid migration, which occurs rapidly and exhibits relatively low adhesion, and mesenchymal migration, which occurs more slowly, has a very high level of adhesion, and is dependent on proteases (64). Both methods are affected by TME’s capacity to support or deter immune cell trafficking, and this capacity can also determine tumour immunological phenotype. Inflamed tumours are characterized by a high density of CD8^+^ T lymphocytes, whereas cold tumours contain an exclusion of these same types of lymphocytes due to the presence of stromal barriers (65).

The development of spatiotemporal models has enabled the simulation of T-cell infiltration patterns based on adhesive protein levels and chemokine gradients. With recent advances in spatial transcriptomics, improved techniques are available to produce high-resolution maps that identify where immune cells are located and how they move between different portions of a given tumour (55). PDE models have been used to predict diffusive migration during hypoxic conditions (35). Hybrid simulation models integrating ODE signalling and ABM migration have been used to predict hotspot regions for T-cell infiltration into solid tumours. ODEs and PDEs are used to model chemokine dynamics at the population level; in contrast, ABMs allow much greater spatial resolution for studying how individual immune cells migrate through heterogeneous TMEs. (66).

### 3.4 Factors affecting immune cell movement in TME

Hypoxia upregulates suppressive chemokines and reduces adhesion molecule expression, impairing trafficking (44). Increased stiffness of the ECM creates a physical barrier to movement while encouraging the development of M2 polarized macrophages (33). The stiffer the ECM is, the less T cell infiltration there is; however, Treg accumulation increases (62). Further limitations on cells’ migration include interstitial flow and solid stress (67). Computational variables that can integrate these variables are needed to understand how these interconnected aspects together hinder immune cell trafficking. In silico models, combining these variables have predicted that modifying the ECM through normalization strategies can increase T cell infiltration. Nevertheless, simulation-derived predictions must be validated using real-life pathological states to be successful in the prognosis and treatment of individuals diagnosed with cancer (67). **Table 2** briefly explains the Biochemical and Biophysical factors of the tumour microenvironment and their Effects on Trafficking and Infiltration.

**Table 2:** Biochemical and biophysical factors of tumour microenvironment and their effects on trafficking and infiltration.

Factor	Description	Effect on trafficking/infiltration
Hypoxia	Stabilizing HIF-1a leads to genetic and metabolic reprogramming (47, 68).	CXCL12 is upregulated due to this phenomenon, resulting in exclusion of CTLs; This effect has a negative impact on CXCL9/10 induction (47) and leads to M2 macrophage polarization.
ECM Stiffness	Collagen deposition and cross-linking are increased (69).	M2-type macrophages act as a physical barrier to facilitate T-cell penetration. Mechanotransduction from these macrophages leads to M2-type polarization through recognition by membrane-associated mechanoreceptors (69).
Interstitial Flow/Solid Stress	The growth of tumours induce mechanical stress and increases Interstitial Fluid Pressure (IFP) (51, 70).	Increased pressure on blood and lymph vessels indicates decreased perfusion of connective tissue, leading to interstitial hypertension. This effect will limit diffusion and the ability of active cells to migrate into the tissue (52)
Chemokine Gradients	There is an imbalance in the quantity of effector-recruiting and immunosuppressive chemokines (71, 72).	CXCL9/10/11 chemokines attract effector T- cells, and CCL2 and CCL22 chemokines recruit Tregs and MDSCs that are suppressive (72).
Adhesion Molecule Expression	ICAM-1 and VCAM-1 expression is downregulated or altered in glycosylation (73).	Affected by the rigidness of the ECM, making it hard for leukocytes to firmly adhere/transmigrate across the endothelium (49).

## 4 In-silico approaches for studying immune–TME interactions

Interactions between immune cells, tumours, and their microenvironments have changed dramatically because we can now use in silico analysis. Multiscale processes involved with the immune system and the solid TME, such as (1) immune cell migration throughout an organism; (2) cytokine signalling; (3) spatial heterogeneity in the immune landscape; and (4) immune cells’ and their associated microenvironments’ response(s) to various therapeutics, make it difficult to experimentally capture all of these interactions. Computational models that pull together data from multiple biological layers, such as genomics, transcriptomics, proteomics, and metabolomics, alongside mathematical predictions, give us a powerful way to make sense of these intricate biological relationships. Ultimately, these tools can help steer and accelerate the drug development process (74).

However, the translational utility of these multi-scale frameworks remains constrained by an ongoing biological fidelity gap, as computational models are often difficult to validate due to a scarcity of high-quality, longitudinal datasets required for parameter calibration and outcome benchmarking. Additionally, finding the right balance is a major limitation (75). **Table 3** provides an overview of in silico approaches for studying immune–TME interactions.

**Table 3:** Overview of in-silico approaches for studying immune–TME interactions.

Approach	Tools and procedures	Strengths	Applications in tumour microenvironment
Computational Modelling	Agent-Based Models (ABM) or Ordinary Differential Equations	Represent Spatial Variability, Chance,	Chemotaxis, Infiltration Barriers,
	(ODE/Partial Differential Equations) Based Method; Hybrid Numerical Simulation Frameworks, such as PhysiCell and CompuCell3D (48, 76)	and Multi-Level Patterns	and Virtual Trials for Immunotherapeutics.
Systems Biology	Quantitative systems pharmacology modelling or integrative multi-omics modelling methods (12, 77)	Quantitative forecasting of collective outcomes of a system	Cytokine networks, checkpoint dynamics, and patient-specific modelling
Network Biology & Pathway Modelling	InnateDB, network analyst, and meta-bridge databases and use of either Boolean or ODE- type methods for signalling networks (78, 79)	Quantifies major individuals and their pathways to influencing other individuals (regulatory, etc.).	Immune signalling, metabolic-immune interactions and target discovery
Machine Learning-Based Predictions	Machine learning methods: random forests, deep neural networks, and graph neural networks (GNNs) or surrogate	Simultaneously represents high volumes of data; quickly provides predictions for	Immune deconvolution, subtype classification and response prediction
	modelling methods (80, 81).	omics/spatial information using high-throughput data analysis (HTA)	

### 4.1 Computational modelling (ABMs, ODEs, PDEs)

Computational models come in many forms, each tailored to capture the behaviour of the immune tumour microenvironment at different levels of complexity and scale (82). ABMs view individual cells as autonomous entities governed by distinct sets of rules, including but not limited to migration, proliferation, apoptosis, and cytokine secretion, that dictate how cells communicate and respond to one another and to their surrounding environment within the spatiotemporal lattice/continuous domain. By capturing these individual behaviours, ABM models are highly efficient at providing insights into the emergence of phenomena associated with immune exclusion, T cell infiltration gradients, and tumour-immune competition (83). Platforms like PhysiCell and CompuCell3D — some already established, others still evolving — offer a hybrid modelling environment that combines ABMs with Partial Differential Equations (PDEs). This pairing enables simulation of how soluble factors, such as chemokines, oxygen, and drugs, diffuse through tissue, providing more realistic portrayals of how chemokine gradients guide cell movement and how the outer matrix acts as a classic barrier to cell trafficking (84). Beyond ABMs and PDEs, Ordinary Differential Equations (ODEs) are also widely used to capture how biological systems change over time, from shifts in cell populations and receptor-ligand binding kinetics to the cascading signals within cells. These equations are often incorporated into Quantitative Systems Pharmacology (QSP) models, which are designed to predict and assess the safety and efficacy of checkpoint inhibitors in virtual clinical trials (85). In contrast to ODEs, which model Temporal Dynamics, PDEs model Spatio-Temporal Transport processes and processes that lead to the formation of Chemokine Gradients, Oxygen Diffusion, and Drug Penetration, which allows for mathematical predictions of the effects of the presence of hypoxia and/or Interstitial Flow on the movements and distributions of Immune Cells (86).

PDEs are particularly effective for modelling continuous fields like nutrient or growth factor diffusion and predicting tumour growth dynamics when spatial and temporal gradients are critical. ODEs are effective for deterministic population dynamics, including tumour-immune interactions or treatment response, where spatial details are less important. ABMs and hybrid models excel at capturing discrete cellular behaviours, spatial interactions with the microenvironment, and phenomena that depend on local heterogeneity (48).

These mechanistic frameworks suffer from intense parameter uncertainty as parameter uncertainty arises when imperfect or sparse experimental data makes it difficult to uniquely estimate model parameters, potentially leading to incorrect biological predictions, and this practical unidentifiability often creates compensating parameter relationships where multiple distinct parameter sets fit the observed data equally well (87). Similarly, parameter identifiability directly determines whether a model’s parameters can be trusted, as unvalidated assumptions or unidentifiable parameters can produce an excellent fit to calibration data while yielding wildly inaccurate predictions in new experimental scenarios and if structural and practical identifiability can result in models relying on indistinguishable parameter combinations that fail to accurately reflect the underlying biology (88).

Models are hard to validate as real-world clinical datasets often lack consistent longitudinal follow-up and are fragmented, or when the data has quality problems like noisy or inconsistent labels, which makes it difficult to notice long-term trajectories or counterfactual outcomes, and additionally, many biological biomarkers cannot be measured continuously, and underlying regulatory networks can change dynamically under therapy (89). The verification of the underlying biological hypotheses and sub-models across diverse patient populations, which requires comprehensive covariates to accurately represent the target population, and, furthermore, their credibility can only be ensured through continuous comparison of in silico predictions with outcomes from retrospective and prospective clinical studies (90).

### 4.2 Systems biology approaches

By combining experimental results and mechanistic models across multiple scales, systems biology aims to develop a quantitative understanding of how the immune system and the TME interact (91). Most importantly, these systems biology approaches emphasise the roles of time, space, and feedback loops in determining the outcome of the immune response and predicting how changes (e.g., immune checkpoint blockade and modulation of cytokine levels) will impact trafficking and tumour control (92). QSP (QSP) models, which are often ODE-based, model the dynamics of entire systems comprising populations of immune cells, soluble mediators, and therapeutic agents, and estimate their parameters using clinical or preclinical data to conduct virtual trials (85). QSP models have also shown promise in forecasting how individual patients might respond to immunotherapy, accounting for the unique characteristics of their TME and the extent to which immune exhaustion mechanisms are at play (93).

The validation status of these systemic models remains weak due to severe inter-patient heterogeneity, as models like QSP typically use generalized parameter distributions to map out broad biological mechanisms since they fail to natively capture real-world individual heterogeneity unless paired with clinical, immunogenomic, or pharmacokinetic data to selectively edit those distributions into distinct virtual patient cohorts (94).

### 4.3 Network biology and pathway modelling

Network biology offers a way to map the complex web of molecular interactions — spanning protein-protein interactions, gene regulatory networks, and metabolic pathways — helping pinpoint the critical nodes and pathways that govern how immune cells communicate and influence one another (95). Examples include databases such as InnateDB, which contain a comprehensive collection of experimentally validated interactions between innate immune genes and proteins, and tools such as Network Analyst, which allow users to visualize and perform a topological analysis of these networks to identify the most important (central) regulators of immune suppression or activation (96). On top of this, MetaBridge enables the integration of metabolomic data into protein-protein interaction networks, shedding light on potential connections between metabolism and immune signalling that could play a meaningful role in reshaping the TME and influencing how cells move through it (97**).** Pathway modelling is a hybrid modelling approach that combines Boolean logic to capture qualitative switch-like behaviours and ODEs to capture quantitative kinetic behaviours (98**).**

A primary limitation of network and pathway modeling is their reliance on static topology and static network models built from genomic data lack spatial context and fail to capture real time, dynamic signal flow, such as post translational protein updates and localized feedback loop and as a result they cannot trace the continuous physical and temporal shifts that determine how a cell respond to its shifting microenvironment (99). In reality, these molecular signalling pathways are highly dynamic. For example, in a tumour, as it progresses, it develops a distinct core-periphery structure, in which the hypoxic core adopts enhanced glycolysis, while the oxygen-rich periphery predominantly depends on oxidative phosphorylation. Under therapeutic stress, this spatial organisation is dynamically remodelled, allowing resistant core cells to integrate both pathways for survival and therapeutic escape (100).

### 4.4 Machine learning-based predictions

Machine learning (ML) leverages large multi-omics datasets and detailed spatial information across multiple dimensions to identify/predict immune infiltration, cell-cell communication, and responses to therapy, without necessarily relying on a known mechanistic model (101). Specific methods of Machine Learning, including random forests, gradient boosting, and deep neural networks, are commonly applied to bulk RNA sequencing data to estimate the proportion of immune cells within tissue samples, or to classify TME subtypes based on how a patient is likely to respond to immunotherapy (102). Machine learning algorithms using advanced neural network methods can also leverage spatial transcriptomics to estimate chemokine-receptor interactions and cell migration trajectories identified through image-based analysis of tissue (103). Overall, there is a need for the use of computer modelling to help understand and identify new treatment options for patients suffering from severe chronic diseases like diabetes and multiple sclerosis (MS). (104). By providing surrogate models that approximate the outputs of expensive agent-based/partial differential equation-based models, machine learning enables the exploration of large parameter spaces using high-throughput methods (105). Continued advances in data-driven predictive analytics will enhance the provision of complementary information to mechanistic-based models when developing personalised immunotherapy strategies (106).

Machine learning predictions are more effective for determining the tissue of origin and histology of tumours, particularly for cancers of unknown primary and poorly differentiated metastases, where immunohistochemistry is unreliable, or the primary site cannot be identified. It is more effective when using whole-genome sequencing rather than exome or targeted gene panels (107).

While machine learning techniques can successfully model complex patterns in large-scale datasets, they are constrained by two key drawbacks: overfitting and limited mechanistic interpretability. Overfitting arises when a data driven machine learning model incorrectly interprets statistical noise as a meaningful signal, effectively memorizing the specifics of a limited training dataset rather than learning broadly applicable patterns and when trained without trained without structural constraints, these models rely on extensive sets of unvalidated genes, resulting in poor mechanistic interpretability and a sharp decline in predictive performance across independent clinical datasets (108).

**Table 4** compares the mechanistic and data-driven in-silico frameworks.

**Table 4:** Comparison of mechanistic and data-driven in-silico frameworks.

Comparative Feature	Mechanistic models	Data-driven models	Ref
Fundamental principle	Capture relevant biophysical processes through kinetic, constitutive and conservation equations.	Reduce system dimensionality and identify key features from high-dimensional signalling data without requiring mechanistic equations, making it ideal for clustering samples and predicting phenotypes at the signal-response level.	(109)
Data Requirement	Require sufficient experimental measurements to properly estimate model parameters, but for high-dimensional systems, mechanistic model parameterisation is often limited by the curse of dimensionality unless many parameters are known.	Requires large amounts of high-dimensional data to infer patterns and achieve accurate predictions	(110)
Biological interpretability	Provides causality and insights into the underlying biological mechanism as they are built on physical laws and causal hypotheses, which enable deductive reasoning and extrapolation beyond observed data	Lacks inherent biological interpretability as they establish statistical correlations rather than causal relationships, making them unable to suggest new treatment protocols, and understanding of mechanistic functions	(111)
Model complexity	Knowledge-driven mechanistic models reduce complexity from the outset by incorporating deliberate, expert-selected simplifying assumptions to ensure the mathematical framework remains tractable, interpretable and explainable	Often employs highly parameterised architectures whose many degrees of freedom allow flexibility to approximate complex relationships, but also create a vague decision-making process, necessitate large amounts of training data and increase vulnerability to overfitting	(112)
Extrapolation	Possess deductive capability, enabling extrapolation to prediction about behaviours not present in the original data, as they are built on mechanistic principles	Have inductive capability as they can only make predictions that relate to patterns within the data supplied, with a limited extrapolation horizon beyond the observed data space	(111)

## 5 Bioinformatics tools and databases used

Advanced bioinformatics technologies create value by providing an extensive array of open-access, standardized, multi-dimensional datasets applicable to validating, performing meta- analysis on, developing hypotheses about, and developing models from biological data (113).

**Figure 3:**
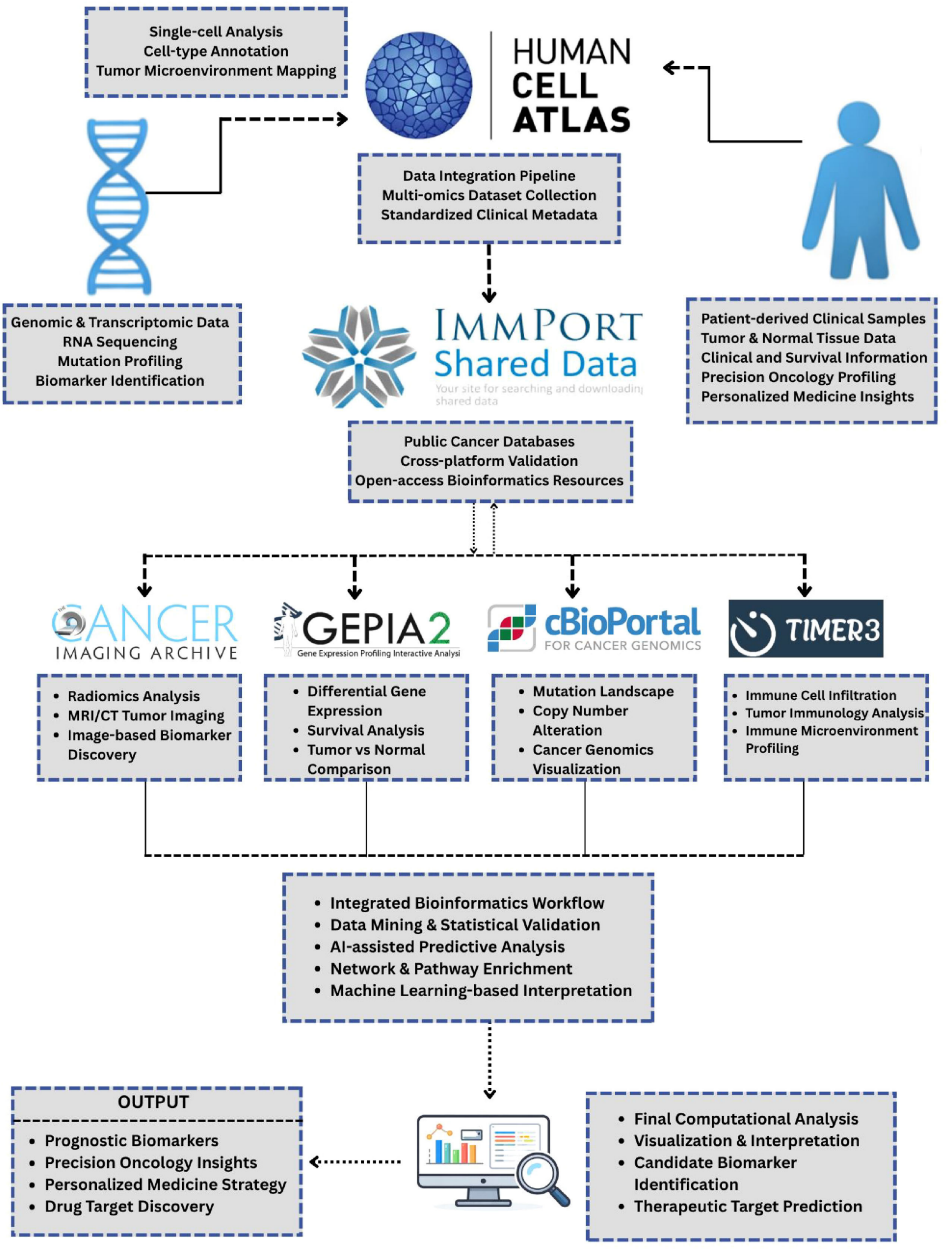
A comprehensive bioinformatics workflow showcasing the obtaining of human cell and genome-based datasets from publicly available data sources, followed by multipurpose analysis for cancer across databases GEPIA2, cBioPortal, TIMER3, and Cancer Imaging Archive, to derive computational interpretations.

Additionally, they create value for the cancer research community by allowing researchers to combine data from several different domains (i.e., clinical, molecular, immunological), thereby providing a platform for researchers to develop reproducible analyses of tumour immune infiltrate; checkpoint mediators, including expression and signaling pathways; the potential for use of chemokine-receptors as therapeutic targets; and predictive capability regarding response to treatment (12). A comprehensive bioinformatics workflow showcasing the obtaining of human cell and genome-based datasets from publicly available data sources, followed by multipurpose analysis was visualised in **Figure 3**.

### 5.1 The cancer genome atlas

The Cancer Genome Atlas (TCGA) began in 2006 as a way to compile and manage an extensive database of human cancer molecular and clinical data, comprising 33 tumour types profiled using various omics technologies (114). TCGA also provides genomic, transcriptomic, proteomic, and clinical data that can be used for in silico exploration of RNA patterns, mutation loads, and immune gene signatures for each cancer type (115). In addition to providing TCGA Data Coordinating Centre data retrieval, TCGA has developed software called TCGA biolinks, which can pre-process, integrate, and reproducibly retrieve data for use in immune–TME interaction and therapy response investigations (116).

### 5.2 Tumour immune estimation resource

TIMER (cistrome.shinyapps.io/timer) is a freely accessible online platform designed for cancer researchers, allowing them to both quantify and visually explore the presence of immune cells within tumours commonly referred to as tumour-infiltrating immune cells (TIICs) by drawing on gene expression data from TCGA (117).

TIMER has developed several deconvolution algorithms capable of estimating the relative abundance of six key immune cell types in tumours: B cells, CD4+ T cells, CD8+ T cells, macrophages, neutrophils, and dendritic cells. Deconvolution-based methods remain sensitive to noise from transcriptional activity and may not accurately delineate rare immune cell populations. Beyond this, it equips researchers with six distinct analytical modules, covering areas such as gene expression, survival, mutation, and somatic copy number alterations Somatic Copy Number Alteration (SCNA) Analysis, Differential Expression Analysis (DiffExp) and Correlation Analysis (117). However, TIMER2.0 has greatly improved TIMER’s algorithms, added user-input capability for gene expression estimation, and converted the interfaces into Shiny-based web applications that will support cross-cancer-type comparisons and the examination of immune cell infiltrates (118).

### 5.3 The cancer immunome atlas

The Cancer Immunome Atlas (TCIA) is an essential tool that examines how cancer immunity has evolved over time using TCGA-derived data. By integrating gene expression profiles, mutation patterns, and immune infiltration signatures, TCIA classifies tumours according to their potential response to immunotherapy and identifies which tumour types respond best to chemotherapy. By studying TCIA, researchers can better understand the relationships between tumour Mutational Burden (TMB), NEO-ANTIGEN load, and immune parameters and clinical outcomes (119).

### 5.4 Tumour and immune system interaction database

TISIDB, developed by Ru et al., is a comprehensive web-based platform that brings together data from TCGA, GEO, and other publicly available databases, providing researchers with a unified space to explore and study the dynamic interactions between tumours and the immune system (120). Through TISIDB, users can study the roles of immune-related genes, molecular pathways, chemokines, checkpoint molecules, and Tumour Infiltrating Lymphocyte (TIL) levels, and correlate genomic changes and methylation with immune infiltration levels. TISIDB also facilitates the comparison of Tumour-Invasive Immune System (TISI) across multiple cancer types, correlating data across multiple Tumour types to identify biomarkers for predicting a response to immunotherapies (120).

### 5.5 Gene expression profiling interactive analysis 2

As reported by Tang et al. (121), GEPIA2 builds on its predecessor, GEPIA, by introducing a range of interactive features that allow users to analyse gene expression patterns and predict clinical outcomes across both cancer and normal tissue samples from the TCGA and GTEx datasets. Key functions of the new platform include an interactive interface for generating plots of differentially expressed genes (like genes upregulated or downregulated in cancer), a correlation matrix for identifying correlations between immune cell markers, and the ability to analyse the relationship between immune cell markers and TME-related genes (121).

### 5.6 cBioPortal for cancer genomics

cBioPortal provides an accessible online resource for viewing multidimensional datasets on cancer genomics, integrating mutation, expression, clone numbers, and other clinical variables from TCGA (The Cancer Genome Atlas) and other databases (122, 123). Within the context of immuno-TME research, cBioPortal provides specific functions that enable researchers to evaluate relationships among immune-gene alterations, checkpoint molecules, treatment response, and survival (123). The cBioPortal API was established to allow users to share their own data, thereby enabling reproducibility in immunogenomic analyses (122).

### 5.7 Human cell atlas

A global project called the Human Cell Atlas (HCA) aims to create a comprehensive reference map of all human cell types using technologies such as single-cell RNA sequencing to capture features at the single-cell level and Spatial Transcriptomics (spT). Using spatially resolved transcriptomic data sets allows better characterisation and identification of specific immune cell locations than standard bulk transcriptomic data. (124). HCA datasets provide high-resolution reference datasets for determining the diversity of immune cell types through normal tissues, and how those cell types are spatially distributed throughout tumours (124). HCA datasets allow identification of immune–stroma interactions, antigen presentation pathways, and mechanisms of immune evasion in tumours (124). The HCA will also provide a reference for validating analyses that move from bulk TCGA/TIMER data to single-cell analyses (124).

A major limitation of bulk repositories is their dependence on bulk tissue transcriptomics. Bulk RNA-seq repositories are inherently limited because they only measure the average gene expression across a blended population of cells. This lack of resolution creates averaging effects that obscure heterogeneity among cell types and allow variations in cellular composition to distort downstream differential expression analysis (125). While computational deconvolution of bulk RNA-seq repositories can more reliably identify major coarse-grained cell lineages, such as total B or T cells, it consistently struggles with fine-grained resolution, often failing to accurately distinguish or sensitively detect highly similar subpopulations and functional states, such as regulatory versus memory CD^4+^ T cells (126). A key disadvantage of bulk RNA deconvolution is its decreased accuracy when mixtures contain a large number of cellular components, especially rare subpopulations or closely related cell types, as well as when the weight matrix is non-orthogonal and the presence of unknown biological content and the choice of measurement scale can significantly affect performance, with most methods failing to accurately estimate rare or highly similar types (127). This limitation is further aggravated in bulk tumour sequencing, where the overwhelming abundance of structural stromal and proliferating malignant cells creates a massive biological background signal that heavily dilutes the weaker signature of infiltrating immune cells, and as a result, this cellular imbalance makes it remarkably difficult for standard deconvolution methods to accurately detect and quantify low-frequency immune subpopulations within the TME (128).

In bulk tissue sequencing, molecular information is averaged across the entire tissue sample, leading to dilution of localized signals generated by infiltrating immune cells by the dominant tumour and stromal populations, and so this masking effect substantially conceals the spatial heterogeneity of the tissue, hiding critical microenvironmental patterns like distinct pockets of T-cell exclusion or localized therapeutic resistance (129). While bulk RNA-sequencing effectively warns broad immune correlates of tumour response, it averages the transcriptional profiles and obscures spatially restricted tumour-intrinsic resistance features. Due to this transcriptional dilution, bulk methods lack the spatial sensitivity needed to identify localised mechanisms of therapeutic evasion, including region-specific cholesterol synthesis pathways that may promote CD8 T cell exclusion (130). The same limitation extends to non-spatial QSP models; while they can match broad, time-dependent bulk metrics, they are insufficient for capturing the localised stochasticity and cellular patterns of the TME. To accurately resolve features such as the geometry of the invasive front, regional T-cell hotspots, or structural fingering patterns, spatially explicit modelling frameworks, such as spatial ABMs, must be utilised that simulate discrete cellular agents interacting within a spatially resolved microenvironment (131).

## 6 In-silico studies on specific immune cells in TME

Computational modelling has been used to analyse the characteristics of immunocytes and their movements (circulating, entering/exiting, or migrating within/out of) the TME, as well as the functional plasticity of each immunocyte type within the TME. By integrating spatial, temporal, and molecular data, we can determine how each of the immunocyte types impacts either anti-tumour immunity and/or immunosuppression (48, 132) and how to design therapeutic interventions that would improve trafficking and function of the immunocyte types capable of inducing cytotoxicity against tumours.

### 6.1 T cells (CD8^+^, CD4^+^, Tregs)

The hostile environment of TME frequently hinders CD8+ T cells’ ability to access and infiltrate tumour tissue, despite being among the most crucial participants in the immune system’s battle against malignancies. Using ABMs and PDEs, researchers have simulated how these cells navigate toward tumours in response to chemokine signals, particularly those generated by CXCL9 and CXCL10. The findings paint a clear picture: regions of dense stromal tissue and a rigid extracellular matrix act as physical roadblocks that impede T cell movement, while low- oxygen conditions suppress CXCR3 expression, further limiting the ability of these cells to home in on their targets (133). When using an ordinary differential equation model in QSP, anti-PD-1 therapy restores CD8+ T-cell migration toward tumours by reducing exhaustion signals and enhancing their response to chemokines (134).

CD4+ helper T cells play a meaningful supporting role in bolstering the activity of CD8+ cytotoxic T cells, largely through the release of cytokines such as IL-2 and IFN-γ. However, the nature of this support depends heavily on the phenotype adopted by the CD4+ T cells. Those that have shifted toward a Th1 phenotype tend to encourage CD8+ T cell migration into the tumour, whereas those that have polarized toward a Th2 or Th17 phenotype instead draw in inhibitory signals that actively obstruct CD8+ T cell movement (135). Tregs also suppress the immune response, and accelerated bio-simulation techniques have revealed that the chemokine CCL22 binds its receptor, CCR4, on Tregs, drawing them into the TME while simultaneously preventing CD8^+^ T cell migration (136). Collectively, these findings point toward a promising therapeutic direction: targeting Tregs through depletion or blocking the CCR4 signalling axis may help restore the capacity of CD8+ T cells to enter the tumour.

### 6.2 NK cells

Natural killer (NK) cells possess the remarkable ability to destroy tumour cells through their innate cytotoxic machinery, without prior exposure or sensitisation to the target. Even in the presence of TME, the combination of TME factors (i.e., chemokine gradients) and trafficking obstacles inhibits NK cells from killing the tumour. The influence of chemokine gradients, such as those driven by CXCL9/10-CXCR3 and CCL5-CCR5 axes, alongside the downregulation of activating receptors like NKG2D and DNAM-1 and adhesion molecules triggered by TGF-β and hypoxia, all shape how NK cells migrate toward, infiltrate, and ultimately eliminate tumour cells. ABM modelling offers a valuable framework for capturing and studying these interconnected dynamics (137).

The predictions of the ABM study suggest that NK cell trafficking to the tumour site with both IL-15 and anti-TGF-β therapy is enhanced by upregulation of chemokine receptors and reversal of anergy (138). Furthermore, the results of the PDE modelling indicate that increased ECM density and elevated interstitial fluid pressure impede NK cell migration; however, the addition of ECM-modifying agents (e.g., hyaluronidase) has been shown to enhance NK cell access to the tumour core (139).

### 6.3 Dendritic cells

Antigen presentation by dendritic cells (DC) and T-cell priming are essential links between innate and adaptive immunity; however, DCs’ ability to traffic into tumours is diminished. Through ODE and network modelling, researchers have been able to simulate the maturation of dendritic cells (DCs) and their navigation along CCL19/21-CCR7 chemokine gradients. These studies have revealed that tumour-derived factors such as PGE2 and IL-10 pose a significant obstacle to DC maturation, suppressing the expression of key chemokine receptors and consequently weakening their ability to travel to draining lymph nodes and carry out effective cross-presentation (77).

ABM studies have further demonstrated that blocking CXCL12 or activating the STING pathway can significantly increase DC accumulation within the tumour. Both approaches work by restoring CCR7 expression, which in turn enhances the priming of CD8^+^ T cells within theTME (140). Finally, PDE-ABM hybrids with spatial modelling have predicted that combining DC-targeted therapy with checkpoint inhibitors enhances infiltration of effector T cells by creating antigen-presenting hotspots (141).

### 6.4 Tumour-associated macrophages

TAMs, based on their action towards the tumour, can be divided into two types. They are named anti-tumour (M1) and tumour-promoting (M2). Regarding immunosuppressive (iTME) cells, M2 TAMs are the most prevalent. The recruitment of TAMs via CCL2–CCR2 and CSF1–CSF1R gradients has been demonstrated in ABM studies (34). These ABMs show TAMs recruited to regions of low oxygen have an increased potential for polarization to an M2 phenotype due to their hypoxic environment and suppression of CD8^+^ T cell infiltration (142).

Using ODE-based models, it has been predicted that inhibiting CSF1R activity or activating CD40 to promote the development of M1 phenotype macrophages will also decrease the ECM matrix remodelling and increase the access of effector cells into tumour regions (143). Network biology analyses of TAMs suggest that pathways involving lactate and PGE2 continue to maintain the M2 phenotype, while targeting these metabolic pathways in M2 TAMs restores their anti-tumour function and enhances TAM infiltration into tumours (144).

### 6.5 Myeloid-derived suppressor cells)

MDSCs are drawn into the TME via CCL2-CCR2 and CXCL12-CXCR4 signalling pathways, where they assume a central role in driving immune suppression (18). PDE-ABM hybrid models demonstrate that in a hypoxic niche, MDSCs form physical and chemical barriers (e.g. ROS, arginase-1, iNOS) that block the recruitment of CD8^+^ T cells and NK cells from the TME to the tumour (145).

Moreover, the QSP modelling suggests that inhibiting CXCR2 or CCR2 may reduce MDSC trafficking, thereby restoring chemokine gradients for effector cell infiltration and enhancing the activity of checkpoint blockade therapies (146). Finally, recently developed machine learning-enhanced simulations combine both single-cell data and QSP models to predict the composition of different MDSC subtypes, as well as their differential impact on MDSC trafficking as a function of different therapeutic modalities (12).

## 7 Computational modelling of immune checkpoints

Computational modelling has become a highly effective method for modelling immune checkpoint pathway dynamics within the TME and predicting how these pathways affect T cell migration, T cell exhaustion, and responses to immunotherapy. Despite their predictive capabilities, ML-based immunotherapies now exhibit overfitting vulnerabilities and reduced interpretability. (147). The models can deliver a summary of how combinations of checkpoint blockade could enhance its effectiveness by simulating the combined effects of molecular kinetics, spatial gradients, and population-level interactions (24).

### 7.1 PD-1/PD-L1

The PD-1/PD-L1 signalling axis is a significant immunosuppressive mechanism in the TME, driving T cell exhaustion and curtailing the ability of anti-tumour T cells to traffic effectively to the tumour (148). Hybrid agent-based and PDE modelling demonstrates that PD-L1 gradients are generated by tumour cells and create exclusion zones where CD8^+^ T cells cannot effectively infiltrate (133). Multiscale modelling approaches incorporating single-cell data have suggested that the therapeutic effectiveness of PD-1 blockade is not universal; rather, it depends on baseline PD-L1 expression on tumour cells and the degree of T-cell infiltration (149). Network models have uncovered a meaningful interplay between the PD-1 and TGF-β signalling pathways, revealing that combining inhibitors of both pathways does a far better job of restoring T cell trafficking than either treatment would on its own (150). PD-L1 upregulation in myeloid/endothelial cells (151) additionally impaired T cell egress using spatially resolved agent-based modelling methods. Spatial transcriptomic techniques provide a more highly resolved description of the location of immune cells associated with checkpoints than bulk transcriptomic datasets. Machine learning-driven quantitative systems pharmacology models have also proven useful in predicting how individual patients are likely to respond to PD-1/PD-L1 antibody therapies, by factoring in each patient’s PD-1/PD-L1 expression levels alongside their tumour mutation burden. Finally, the predictive accuracy of immune checkpoint models remains reliant on variable quantification, biological assumptions, and external clinical validation. (152). Furthermore, the effects of PD-1 bispecific on the efficacy of checkpoint blockade have been analysed and reported using Kinetic Monte Carlo simulation models (153).

### 7.2 CTLA-4

CTLA-4 inhibits T cell activation and trafficking via competitive inhibition of CD28-B7 interactions (CD80/CD86) on antigen-presenting cells and blocks co-stimulatory signals and transmits inhibitory signals that restrict the activation of T cells (154). ABM simulations have shown that blocking CTLA-4 improves the capacity of CD8+ T cells to migrate toward developing tumours, largely by relieving Treg-mediated suppression and making chemokine gradients more accessible (155). Complementing this, systems pharmacology models provide evidence that simultaneously targeting both CTLA-4 and PD-1 pathways produces a synergistic effect on T cell trafficking that neither target alone can fully achieve (156). Network biology analyses indicate that inhibition of CTLA-4 increases IL-2 and IFN-γ, which stimulates Th1 polarization and the recruitment of effector cells (148). Spatio-temporal PDE-ABM hybrid models predict that CTLA-4 blockade is most effective in the context of early-stage tumours with a high degree of antigen presentation (157). Machine learning models trained on clinical trial data can predict CTLA-4 blockade responsiveness based on baseline T cell infiltration and B7 expression (102). Finally, kinetic models have enabled precise quantification of how ipilimumab influences T cell trafficking dynamics and alleviates T cell anergy within the TME (158).

### 7.3 Emerging checkpoints

The impact of newly identified immune checkpoint proteins — including LAG-3, TIM-3, TIGIT, VISTA, and BTLA — on T cell exhaustion and migratory capacity has been explored through a range of computational and experimental models (159). An ODE model studying LAG-3 predicts that LAG-3 binding to MHC-II enhances CD8^+^ T cell proliferation and post-activation migration. The model further identified that MHC class II^+^ and PD-1^+^ cells share overlapping inhibitory signalling pathways (153). ABM simulations exploring the relationship between TIM-3 and galectin-9 have further revealed that blocking TIM-3 triggers a marked surge of NK cells and CD8+ T cells into oxygen-depleted tumour regions, effectively reversing the state of terminal exhaustion in these cells (159). In terms of dual blockade of TIGIT/CD155 and CD112R/CD112, network modelling of these interactions shows that blockade of either TIGIT or PD-1 alone produces a lesser effect on T cell and NK cell trafficking than does combined blockade (160).

The blockade of VISTA was modelled, and the results indicated that VISTA blockade increases effector cell migration into immunosuppressive TME niches by reducing myeloid-mediated inhibition (161). The interactions between BTLA and HVEM were also simulated using kinetic models, demonstrating that BTLA blockade reinvigorates early T cell activation and chemokine responsiveness (162). In the last decade, several multiscale hybrid models have developed new combinatorial strategies that utilise immune checkpoint proteins in conjunction with PD-1 and CTLA4; these models predict that the synergy of the combination blockade would have greater effects on T cell and NK cell trafficking than single blockade with either immune checkpoint protein (156). Finally, machine learning methodologies have been developed to establish patient-specific immune checkpoint expression pattern profiles, further refining predictions of the likelihood of successful combination therapy. Graph-based computing techniques and GNNs have enabled improved modelling of the structure of communication between the spatial distribution of immune cells and networks of checkpoint interactions in TMEs. (152).

### 7.4 Predictive models for response to immunotherapy

Immunotherapy responses can be predicted using predictive models based on Combined Immune Tumour Mutation Burden (MI) checkpoints, Immune Infiltration (TD), and TME features (163). QSP models calibrated against clinical trial data have shown that patients who respond favourably to therapy tend to share certain features: a higher abundance and greater population of naïve CD8+ T cells within the tumour, coupled with a lower ratio of Tregs and MDSCs (149). Machine Learning Classifiers trained on both TCGA and Single-Cell Clone Sequencing Data are being used by researchers in an effort to classify and profile patients as responders or nonresponders based on their Checkpoint Expression, the Type of Chemokine Signature Present in the TME and the Number of Neoantigens Available to Each Patient (102). Predictions based on ABMs of combination anti-PD-1/CTLA-4 blockade indicate that this dual therapy achieves the highest response rates when PDL-1 and CTLA-4 are distributed throughout the tumour, with the greatest responses observed in inflamed tumour regions (156). Through network modelling, researchers have assembled panels of biomarkers — encompassing PD-L1, LAG-3, and an interferon-gamma (IFN-γ) gene signature, alongside key chemokines — that have proven reliable indicators of which patients are most likely to benefit from combined PD-1 and CTLA-4 blockade therapy (148). Space-Time and PDE Model Predictions illustrate the Effects of Hypoxia and ECM rigidity on trafficking patterns and response rates (133). Deep Learning Models relied upon spatial transcriptomics to map location and identify active hotspots resulting from PD-1 and PD-L1 Interactions (164). Hybrid Machine Learning and Mechanistic Models allow the incorporation of Multi-Omics Data, enabling the Personalisation of Immunotherapy Protocols and the Discovery of Mechanisms of Resistance (152).

## 8 Multi-omics integration in in-silico TME studies

Integrating multiple types of Omics information is central to all computer-based studies on TME. Using multi-omics, scientists can combine multiple layers of molecular information to digitally reconstruct immune cell movement, how they communicate with other cells, and to predict their response to different treatments. Using multi-omics methods can address some of the issues faced by traditional single-Omics methods, such as identifying how the tumour’s environment can confer new properties that affect a tumour’s responsiveness or resistance to immunotherapy (91, 165).

### 8.1 Genomics + transcriptomics integration

Comparing genomic and transcriptomic data side by side provides deeper insight into how somatic mutations, copy number alterations (CNAs), and neoantigens relate to one another through gene expression patterns. When these two analytical approaches are combined, they become particularly powerful at unpacking the various factors that shape the recruitment and movement of immune cells. For example, combining Weighted Gene Co-expression Network Analysis (WGCNA) with TCGA somatic mutation data reveals how specific gene modules linked to immune function drive CD8^+^ T cell recruitment and chemokine expression. This combined approach also deepens our understanding of how genomic instability erects barriers that restrict different lymphocyte populations from entering the TME (166). When transcriptomic deconvolution techniques are paired with somatic mutation analysis, it becomes possible to appreciate how significantly certain driver mutations, such as KRAS and TP53, influence the tumour’s immune landscape. Specifically, these mutations appear to hinder the recruitment of effector T cells into the TME while simultaneously creating conditions that favour the influx of MDSCs (167).

Furthermore, combining neoantigen prediction workflows with RNA-seq deconvolution techniques enables measurement of the extent to which mutation-derived neoepitopes shape the ability of CD8+ T cells to home to the tumour and mount a meaningful response to immune checkpoint blockade therapies (168). Lastly, applying Multi-Omics Factor Analysis (MOFA) to whole exome sequencing and bulk RNA-seq data can uncover hidden factors that link genomic instability to immunosuppressive states within the TME, while also offering valuable insight into why CD8+ trafficking of T cells to the tumour site becomes compromised (169). Utilizing a method called graph-based deep learning, researchers have been able to analyze genomic and transcriptomic data together (impaired immune infiltration and/or traffic flow) to identify different immune infiltration subtypes across various types of cancers (102). Using joint genomic-transcriptomic latent variable modelling, copy number alterations of specific genes encoding chemokine receptors (such as CXCR3 or CCR5) can also be investigated and affect patterns of immune infiltration (170). The modernization of deep learning approaches that combine mutation profiling with expression matrices has enabled research to identify predictive signatures associated with restoring immune responses. Therefore, previous studies demonstrate that this approach is effective at predicting the number of patients who will respond to PD-1 or PD-L1 blockade (152).

Bulk RNA-sequencing and genomic data can estimate the overall abundance of T cells in a sample, they are largely insensitive to spatial microarchitecture and consequently, bulk methods cannot distinguish between and inflamed phenotype, where T cells actively infiltrate and co-localize with tumour cells and an excluded phenotype, where immune cells are physically and chemokine mediated restricted to the surrounding stroma, failing to penetrate the tumour (171). Bulk genomic sequence also lacks spatial context; it cannot identify the structural stroma or active molecular pathways, such as TGFβ signalling, that trap immune cells outside the epithelium and drive an excluded phenotype, as high genetic mutational or neoantigen burden typically correlates with increased T cell numbers overall. Genomic profiling alone is deficient in predicting whether those T cells will successfully infiltrate the tumour (172).

### 8.2 Proteomics & metabolomics

Bringing proteomics and metabolomics together offers a richer picture of how the TME shapes the movement and function of immune cells, particularly T cells, as they mature into fully active effector cells. Methods such as reverse-phase protein array (RPPA) and metabolomic profiling enable identification of co-regulated proteins and metabolites generated through the IDO1-kynurenine pathway, which can suppress CD8+ T cell migration by depleting available tryptophan (173). The results of a comprehensive analysis using untargeted metabolomic profiling combined with mass spectrometry-based proteomic analyses demonstrate that M2 TAMs prevent the infiltration of NK cells and T cells into the TME by increasing lactate levels, which act as a metabolic barrier (174). Lastly, latent factors that correlate with the glucose metabolic pathway and the production of immunosuppressive chemokines, CCL2 and CXCL12, which are known to promote MDSC infiltration, were identified using the MOFA+ (Multi-Omics Factor Analysis) analysis of combined metabolomic and proteomic datasets (175). The combination of spatial metabolomics with proteomics has established an evidence base for the determination of lactate and kynurenine gradients in the hypoxic regions of the TME, both of which create barriers to the migration of activated effector T cells (176). The use of functional genomics in conjunction with proteomic/metabolomic assessments has revealed multiple kinase - metabolite “hubs” that are critical for the definition of M2 polarization and that inhibit T cell infiltration (170).

The functional turnover of cellular metabolites occurs almost instantly, whereas mRNA transcription and degradation operate on a vastly slower timeline. Standard ordinary differential equation models are challenged by this extreme timescale separation, which is mostly addressed through advanced computational frameworks such as Differential-Algebraic Equations (DAEs) to bridge the temporal gap without numerical instability (177).

### 8.3 Single-cell and spatially resolved bioinformatics approaches

The benefits of specialised transcriptomics and scRNA-seq help us create maps of immune cells within the TME, enabling us to accurately model their interactions with other cells and their migration. When scRNA-seq data is deconvoluted with spatial data, it helps create a better understanding of how chemokine gradients and networks of cell–cell interactions lead to the exclusion or infiltration of CD8^+^ T cells (174). Spatial transcriptomics platforms, such as MERFISH and Visium, in conjunction with scRNA-seq, illustrate that effector T cells and NK cells cannot readily enter immunosuppressive microenvironments characterised by high concentrations of PD-L1, TGF-β and lactate (178, 179). Beyond this, leveraging cell-to-cell communication tools such as CellChat, along with ligand-receptor inference frameworks applied to spatially resolved single-cell RNA sequencing datasets, allows researchers to pinpoint the specific ligand-receptor interactions that drive MDSC recruitment and promote the buildup of Tregs within the TME (180, 181). Using graph-based deep learning models for spatial transcriptomics enables researchers to infer the trafficking trajectories of each cell type and to reveal which areas of tumours have the highest levels of contact between different cell types (182). By integrating multi-modal datasets from scRNA-seq, spatial proteomics, and metabolomics, researchers have also gained insights into how metabolic activity within a given region affects immune trafficking (176). Even with these advances in high-resolution imaging and methods for analysing multi-omics datasets, integrating datasets remains challenging, particularly due to batch effects, heterogeneity in data types, and differences in resolution across platforms.

Despite the resolution provided by these single-cell and spatial platforms, integrating them across distinct patient cohorts presents major technical integration challenges as single cell platforms like scRNA seq suffer from high technical variation, including higher dropout rates, lower RNA input, high cell to cell variations and a higher proportion of zero counts, making batch effects much more severe than in bulk technologies (183). Similarly, the computational biology community lacks established criteria to evaluate the effectiveness and usability of single-cell, multi-omics analysis techniques, despite their widespread demand. Since ground truth is rarely available, benchmarking techniques for multimodal data are intrinsically challenging. There are still many unidentified methods by which molecular and cellular processes interact across scales. By simulating high-throughput data in silico, ground truth can be introduced to evaluate computing performance in well-defined data integration tasks. However, modelling data across modalities in the context of data integration makes it more difficult to simulate a realistic covariance structure among features (184).

## 9 Applications in cancer immunotherapy research

In silico methods have evolved from prototyping or modelling of the TME to precise, personalized applications in the immunotherapy of cancer (132). Simulation of trafficking of immune cells and checkpoint dynamics, along with interactions of multi-omics with the drug effects, provides insight into how the treatment will respond to the body environment and optimizes the combination patterns, which leads to the design of personalized therapy and tends to learn more about the mechanism of resistance (147).

### 9.1 Predicting treatment response

Bioinformatics-based computational models predict immunotherapy response by combining immune infiltration scores, checkpoint expression, tumour mutation burden (TMB), and other TME features (12). QSP models are designed to calibrate this clinical data to analyse anti-PD-1/PD-L1 response based on baseline CD8^+^ T cell density, PD-L1 expression, and MDSC/Treg ratios (134). Deep learning models for spatial transcriptomics help model PD-L1 and chemokine gradients to predict the location and type of tumour responding to checkpoint blockade (164). Hybrid ABM–QSP simulation helps predict density, and hypoxia-induced exclusion reduces response rate, while combining that data with agents targeting angiogenesis improves the final outcome (133).

To identify gene modules (IFN-γ, CXCL9/10, GZMB) that correlate with complete immunotherapy responsiveness, network-based models will be used (148). Additionally, multi-task deep learning fragments will combine bulk RNA sequencing data and mutation data to differentiate responders and non-responders across various cancer types (152). To predict responses from single-cell data, graph attention networks are applied by replicating interactions between each cell type checkpoint and trafficking bottlenecks. Despite these advances, predictive models for immunotherapy are still restricted by the large amount of variability between patients (inter-patient heterogeneity), uncertainty regarding parameters used to construct the model, and a lack of substantial external clinical validation. (181).

### 9.2 Personalized immunotherapy modelling

Patient-specific in silico models for immunotherapy use multiomics technology to simulate the TME dynamics (185). Digital twin models will also be constructed from scRNA-seq and spatial transcriptomics data derived from patients, which will help simulate virtual tumours to test PD-1 blockade and CTLA-4 inhibition, which precede infiltration, restoration of an individual, and the benefit of survival (186). QSP models that incorporate individuals’ PD-L1 expression and T cell infiltration can predict response probability and guide dosage scheduling (134). To reconstruct chemokine gradients and stromal barriers, single-cell-informed ABMs were used to predict the patient’s benefit from blocking to enhance trafficking (174).

Precision immunotherapy is now advancing through several complementary approaches. First, multi-omics factor analysis (MOFA) applied to patients with matching data will help personalise the immunotherapy targets (169). Second, digital twins derived from deep learning models will integrate various omics data to simulate CAR-T Cell trafficking (23). Third, Bayesian inference models will update the personalised parameters, such as checkpoint density, during the period of therapy to adjust the regimens (134), and finally, patient-derived organoids can be integrated with computational models to predict the response to antibodies and combinations in the checkpoint (141).

### 9.3 Simulation of drug–TME interactions

There are computational models for simulating drug activity with the TME, which can provide insight into the immunotherapy pharmacology from multiple perspectives, including pharmacodynamics, pharmacokinetics, and the spatial effects of immunotherapies in heterogeneous tumours (24). PhysiCell-based agent-based model (ABM) simulations of the anti-PD-1 antibody demonstrate that increasing tumour interstitial fluid pressure limits the antibody’s penetration and effectiveness (133). Additionally, PDE-ABM hybrid simulations have revealed that anti-CTLA-4 antibody treatment disrupts the interaction between Tregs and dendritic cells, ultimately enhancing the gradients that drive CD8+ T cell infiltration into the tumour (150). Finally, QSP simulations of CAR-T cell therapy shed light on how this treatment influences cytokine release syndrome, its overall effectiveness, and its impact on immune cell trafficking. These models also help identify optimal dosing strategies that strike the right balance between maximising therapeutic benefit and minimising the risk of toxicity (152). Cell-based ABMs will simulate the diffusion of anti- PD-1 antibody that binds to PD-1 on T cells, thereby restoring the trafficking, ultimately proving that interstitial pressure in tumours is responsible for limiting the penetration and efficacy of the drug (133).

## 10 Challenges and limitations of in-silico approaches

Despite considerable progress in this field, the use of bioinformatics approaches to study immune cell trafficking and interactions within the TME is still constrained by limited patient cohorts and wide variability across datasets (6). These limitations will be due primarily to the following factors: data quality; model complexity (error); temporal and spatial resolution; and biological fidelity (48). As a result, in silico investigations into how immune cells migrate to and interact with the TME have suffered from low accuracy and limited predictive power compared with animal models (132).

In addition to low data integrity, multi-omic datasets containing well-annotated cellular populations derived from diverse sources are in very short supply, particularly for rare cell populations or transiently evolving cellular processes (175). Furthermore, datasets derived from single-cell RNA-seq and spatial transcriptomic studies are often affected by one or more of the following problems: batch effects, missing values, and low signal-to-noise ratio (187). All these issues result in biased estimates of gene expression deconvolution and cellular communication (188).

Most importantly, very few available databases contain spatial and functional information in a way that allows them to be compared, which limits the modelling capabilities for chemokine gradients, adhesive interactions, and transport across physical barriers (178). For the rarest cell populations, standardised, high-quality data are less readily available (141). Furthermore, when single-cell RNA sequencing and spatial transcriptomics data are of low quality, issues such as missing or mismatched values or low resolution can lead to biased predictions of immune cell trafficking (189). Consequently, data sets that lack functional and precise information will negatively impact the accuracy of chemokine gradients and adhesion dynamics (186).

ABMs, ODE/PDE hybrids, and QSP frameworks often involve dozens to hundreds of parameters, but these parameters are often poorly constrained by data because there are few experimental measurements to guide the estimates (6). Sensitivity analyses demonstrate that small changes in less well-constrained parameters (e.g., chemokine diffusion coefficients, integrin binding kinetics) can completely alter the predicted patterns of infiltration and the resulting therapies (48). Machine learning models are also frequently over-fitted when trained on small sample sizes, leading to limited generalizability across different cancer types or patient populations (102).

ABM-like agent-based models and hybrids will require hundreds or even thousands of parameters, but many of these models will lack accurate experimental data (111). Sensitivity analysis reveals that even small changes in these parameters, such as chemokine diffusion coefficients and binding kinetics, can rapidly alter infiltration patterns, thereby affecting the outcome (48). Also, overfitting is more common in ML models, which can reduce generalizability across various cancer types and populations (185).

Validation against clinical and experimental outcomes is frequently inadequate (111). Many models are calibrated to bulk TCGA data or preclinical mouse models but fail to reproduce human trial results due to inter-species differences in TME architecture, immune composition, and pharmacokinetics (186). Prospective validation in independent cohorts is rare, and few models have undergone rigorous external validation for response prediction or trafficking restoration (163). Validation against these outcomes is often inadequate, as many models are calibrated on bulk TCGA data and frequently fail to replicate results in human trials due to mismatches in TME architecture, immune system composition, and pharmacokinetics (48). While surrogate machine learning models can address some challenges, they risk sacrificing mechanistic interpretability (111). Furthermore, cost and scalability issues continue to limit the exploration of these parameters and the opportunity to combine these therapies (132). High-fidelity multiscale simulation requires significant resources, ultimately limiting the use of personalised digital twins or virtual screening (185). Surrogate machine learning models also help but carry the risk of losing the mechanistic interpretability (111).

## 11 Future directions

The computational study of immune cell trafficking and the tumour microenvironment is a rapidly advancing field, propelled forward by developments in artificial intelligence, single-cell omics technologies, and increasingly sophisticated computational frameworks backed by experimental validation (147, 190). Looking ahead, the field will largely focus on tackling persistent challenges in precision, resolution, and the interpretability of complex model outputs.

AI-driven immune modelling is now advancing through the integration of fundamental models with large-scale multi-omics data, including transcriptomics, proteomics, metabolomics, etc. (191). All of these will be combined to predict immune trafficking at an unpredictable resolution. For instance, tools such as graph neural networks will learn to represent TME cell-to-cell communications, enabling zero-shot prediction of interactions between novel chemokine receptors and cross-linking checkpoints (81). Generative AI will synthesize artificial datasets to manage problems such as limited clinical samples and overfitting in rare types of tumours (192). Some AI methods, such as SHAP and attention mechanisms, can credibly enhance the interpretation of such predictions, revealing whether trafficking is being restored or evaded (193).

Digital twins of cancer, where patient-specific virtual replicas of TME are emerging as a transformative approach, will continuously integrate clinical data from imaging and multi-omics to stimulate real-time immune cell trafficking and predict individualised responses to therapy (194). Certain limitations exist when using computer models to elucidate the causes of these diseases; however, significant progress has been made in applying these approaches to enhance current understanding of disease pathophysiology and to better understand how new therapies work. (195). Hybrid frameworks combining digital twins, machine learning, and deep learning will enable high-throughput virtual trials, facilitating combination immunotherapies and optimising patient-specific dosing regimens (80). This collaborative framework will help develop digital twin technology and preserve patients’ privacy (196). A deep learning computational toolkit that integrates single cell RNA sequencing, bulk transcriptomics and spatial transcriptomics to identify and prioritise clinically relevant spatial niches within TME using a relative expression ordering transformation is TiRank, which enables the identification of phenotype-associated cell subpopulations and spatial niches without requiring matched single cell labels for training (197).

Integrating subcellular, cellular, and tissue-level models will enhance the ability to stimulate the TME with spatial and temporal fidelity (48). For example, in the three-dimensional TME structure, drug interactions and penetration were captured using diffusion- limited reaction-transport models (139). Tools such as time-series, single-cell, and spatial data will help simulate trafficking trajectories dynamically. Such approaches will facilitate the analysis of the resistance adaptation and response over the months (198). Complementing these dynamic simulation approaches, an emerging technology is the development of whole slide foundation models (TITAN), which encode entire whole slide images into general purpose feature representations using vision language pretraining on millions of images and pathology reports which enables off the shelf applications like zero shot diagnosis, cross modal retrieval, and rare cancer retrieval, without task specific fine tuning, which offers a powerful, scalable extension to trajectory-based resistance and response modeling (199). Another major emerging technology is spatial multi-model integration frameworks which moves beyond single modality analysis to unify pathology image embeddings with spatially resolves transcriptomic and multi-omics data (200). Complementing these static spatial integration approaches, technologies such as Zman-seq, a longitudinal labelling method that uses time-stamped fluorescent antibodies to track immune cell state transitions over time, and intraviral imaging techniques, for example, imaging windows for real-time spatiotemporal recording of cell movement and interactions in live animals, add critical temporal dynamics. Computational tools like MOSCOT and SLAT enable the study of dynamic tissue organization as they are being developed to align and integrate spatial transcriptomic data at multiple time points (201).

For in silico evidence to be accepted and translated into clinical practice, proper validation, clinical trials, and a regulatory framework are needed (202). To accelerate regulatory approval of these immunotherapies, virtual patient cohorts will be generated from digital twins (203).

These future implications will ultimately transform in silico immune-oncology from a traditional approach to a predictive, precise, and personalised platform, thereby improving immunotherapeutics for many types of cancer.

## 12 Discussion

The TME is now becoming a decision maker of progression of cancer, immune evasion, and immunotherapy responses, slightly shifting from seeing the cancer as the only cause for autonomous cell disease to an ecosystem that is mostly guided by immune tumour interactions (25, 26). Trafficking of immune cells, which is largely mediated by chemokine gradients and adhesive molecules, plays a significant role in maintaining the balance between immunity against tumours and immune suppression (18, 54). Bioinformatics approaches have demonstrated that advances in this field often yield multiscale simulations that combine molecular, cellular, and tissue-level dynamics, surpassing traditional methods (6, 132).

Computational modelling tools, including agent-based and hybrid frameworks, have shed light on how particular populations of immune cells, such as T cells and dendritic cells, shape the tumour microenvironment, uncovering the underlying mechanisms that govern immune cell recruitment and the deployment of functional effectors (55, 204). Network models and systems biology helped identify checkpoint Pathways and their relationships with metabolic and stromal cues, ultimately enabling us to predict therapeutic opportunities (205, 206). Also, multi-omics integration provides a combined reconstruction of TME networks, revealing factors responsible for immune response and trafficking (91, 169).

Tools and databases in modern bioinformatics provide full access to advanced, high- resolution data, which ultimately makes it easier to analyse and test hypotheses across various cancer types (12). Even though the advantages are huge, some challenges still persist. Challenges include parameter uncertainty, resolution issues, overfitting, improper validation, and the compounded biological systems, which continue to limit the precision and accuracy of today’s advanced models (111, 207). The modern bioinformatics field aims to address these limitations by creating standardised datasets, introducing machine learning prototypes, and ensuring proper validation, all of which need to be combined for future progress (208).

Towards the future, AI-powered models, personalised therapeutics, and regulatory validation of this in silico evidence will surely transform computational immuno-oncology into a precise, personalised, and real-time tool. Lastly, translating data from simulation-based predictions into patient-based clinical applications remains a significant challenge for practitioners in computational oncology. (208). Ultimately, these advancements should take responsibility for overcoming resistance to immunotherapies, guiding therapeutic strategies and delivering personalised healthcare tailored to each individual’s unique immune landscape. By integrating AI-driven modelling, multi-omics data, and rigorous validation, computational immune oncology can become a precision tool that improves cancer patients’ outcomes.

## References

[1] Wang Q Shao X Zhang Y Zhu M Wang FXC Mu J . Role of tumour microenvironment in cancer progression and therapeutic strategy. Cancer Med. (2023) 12:11149–65. doi: 10.1002/cam4.5698 36807772 PMC10242329

[2] Yang K Sheng Y Huang C Jin Y Xiong N Jiang K . Clinical characteristics, outcomes, and risk factors for mortality in patients with cancer and COVID-19 in Hubei, China: A multicentre, retrospective, cohort study. Lancet Oncol. (2020) 21:904–13. doi: 10.1016/S1470-2045(20)30310-7 32479787 PMC7259917

[3] Bader JE Voss K Rathmell JC . Targeting metabolism to improve the tumour microenvironment for cancer immunotherapy. Mol Cell. (2020) 78:1019–33. doi: 10.1016/j.molcel.2020.05.034 32559423 PMC7339967

[4] Anderson NM Simon MC . The tumour microenvironment. Curr Biol. (2020) 30:R921–5. doi: 10.1016/j.cub.2020.06.081 32810447 PMC8194051

[5] Salmon H Franciszkiewicz K Damotte D Dieu-Nosjean M-C Validire P Trautmann A . Matrix architecture defines the preferential localization and migration of T cells into the stroma of human lung tumours. Cancer Res. (2012) 72:5180–9. doi: 10.1172/JCI45817 22293174 PMC3287213

[6] Metzcar J Wang Y Heiland R Macklin P . A review of cell-based computational modeling in cancer biology. JCO Clin Cancer Inf. (2019) 3:1–13. doi: 10.1200/CCI.18.00069 30715927 PMC6584763

[7] Finotello F Trajanoski Z . Quantifying tumour-infiltrating immune cells from transcriptomics data. Cancer Immunol Immunother. (2018) 67:1031–40. doi: 10.1007/s00262-018-2150-z 29541787 PMC6006237

[8] Newman AM Liu CL Green MR Gentles AJ Feng W Xu Y . Determining cell type abundance and expression from bulk tissues with digital cytometry. Nat Biotechnol. (2019) 37:773–82. doi: 10.1038/s41587-019-0114-2 31061481 PMC6610714

[9] Yoshihara K Shahmoradgoli M Martínez E Vegesna R Kim H Torres-Garcia W . Inferring tumour purity and stromal and immune cell admixture from expression data. Nat Commun. (2013) 4:2612. doi: 10.1038/ncomms3612 24113773 PMC3826632

[10] Becht E Giraldo NA Lacroix L Buttard B Elarouci N Petitprez F . Estimating the population abundance of tissue-infiltrating immune and stromal cell populations using gene expression. Genome Biol. (2016) 17:218. doi: 10.1186/s13059-016-1070-5 27765066 PMC5073889

[11] Junttila MR de Sauvage FJ . Influence of tumour micro-environment heterogeneity on therapeutic response. Nature. (2013) 501:346–54. doi: 10.1038/nature12626 24048067

[12] Thorsson V Gibbs DL Brown SD Wolf D Bortone DS Ou Yang TH . The immune landscape of cancer. Immunity. (2018) 48:812–830.e14. doi: 10.1016/j.immuni.2018.03.023 29628290 PMC5982584

[13] Nguyen H Nguyen H Tran D Draghici S Nguyen T . Fourteen years of cellular deconvolution: methodology, applications, technical evaluation and outstanding challenges. Nucleic Acids Res. (2024) 52:4761–83. doi: 10.1093/nar/gkae267 38619038 PMC11109966

[14] Cobos FA Panah MJN Epps J Long X Man TK Chiu HS . Effective methods for bulk RNA-seq deconvolution using scnRNA-seq transcriptomes. Genome Biol. (2023) 24:177. doi: 10.1186/s13059-023-03016-6 37528411 PMC10394903

[15] Baghban R Roshangar L Jahanban-Esfahlan R Seidi K Ebrahimi-Kalan A Jaymand M . Tumour microenvironment complexity and therapeutic implications at a glance. Cell Commun Signaling. (2020) 18:59. doi: 10.1186/s12964-020-0530-4 32264958 PMC7140346

[16] Xiao Y Yu D . Tumor microenvironment as a therapeutic target in cancer. Pharmacol Ther. (2021) 221:107753. doi: 10.1016/j.pharmthera.2020.107753 33259885 PMC8084948

[17] Qin H Sheng W Zhang G Yang Q Yao S Yue Y . Comprehensive analysis of cuproptosis-related prognostic gene signature and tumour immune microenvironment in HCC. Front Genet. (2023) 14:1094793. doi: 10.3389/fgene.2023.1094793 36891150 PMC9986498

[18] Nagarsheth N Wicha MS Zou W . Chemokines in the cancer microenvironment and their relevance in cancer immunotherapy. Nat Rev Immunol. (2017) 17:559–72. doi: 10.1038/nri.2017.49 28555670 PMC5731833

[19] Novysedlak R Guney M Al Khouri M Bartolini R Koumbas Foley L Benesova I . The immune microenvironment in prostate cancer: a comprehensive review. Oncology. (2025) 103:521–45. doi: 10.1159/000541881 39380471 PMC12140600

[20] Yankeelov TE Quaranta V Evans KJ Rericha EC . Toward a science of tumor forecasting for clinical oncology. Cancer Res. (2015) 75(6):918–23. doi: 10.1158/0008-5472.CAN-14-2233 25592148 PMC4359948

[21] Galon J Bruni D . Tumour immunology and tumour evolution: intertwined histories. Immunity. (2020) 52:55–81. doi: 10.1016/j.immuni.2019.12.018 31940273

[22] Chen DS Mellman I . Elements of cancer immunity and the cancer–immune set point. Nature. (2017) 541:321–30. doi: 10.1038/nature21349 28102259

[23] Kather JN Pearson AT Halama N Jäger D Krause J Loosen SH . Deep learning can predict microsatellite instability directly from histology in gastrointestinal cancer. Nat Med. (2019) 25:1054–6. doi: 10.1038/s41591-019-0462-y 31160815 PMC7423299

[24] Ribas A Wolchok JD . Cancer immunotherapy using checkpoint blockade. Science. (2018) 359:1350–5. doi: 10.1126/science.aar4060 29567705 PMC7391259

[25] Hanahan D . Hallmarks of cancer: New dimensions. Cancer Discov. (2022) 12:31–46. doi: 10.1158/2159-8290.CD-21-1059 35022204

[26] Quail DF Joyce JA . Microenvironmental regulation of tumour progression and metastasis. Nat Med. (2013) 19:1423–37. doi: 10.1038/nm.3394 24202395 PMC3954707

[27] Hanahan D Coussens LM . Accessories to the crime: Functions of cells recruited to the tumour microenvironment. Cancer Cell. (2012) 21:309–22. doi: 10.1016/j.ccr.2012.02.022 22439926

[28] Binnewies M Roberts EW Kersten K Chan V Fearon DF Merad M . Understanding the tumour immune microenvironment (TIME) for effective therapy. Nat Med. (2018) 24:541–50. doi: 10.1038/s41591-018-0014-x 29686425 PMC5998822

[29] Gajewski TF Schreiber H Fu YX . Innate and adaptive immune cells in the tumour microenvironment. Nat Immunol. (2013) 14:1014–22. doi: 10.1038/ni.2703 24048123 PMC4118725

[30] Kalluri R . The biology and function of fibroblasts in cancer. Nat Rev Cancer. (2016) 16:582–98. doi: 10.1038/nrc.2016.73 27550820

[31] Jain RK . Antiangiogenesis strategies revisited: From starving tumours to alleviating hypoxia. Cancer Cell. (2014) 26:605–22. doi: 10.1016/j.ccell.2014.10.006 25517747 PMC4269830

[32] Egeblad M Nakasone ES Werb Z . Tumours as organs: Complex tissues that interface with the entire organism. Dev Cell. (2010) 18:884–901. doi: 10.1016/j.devcel.2010.05.012 20627072 PMC2905377

[33] Pickup MW Mouw JK Weaver VM . The extracellular matrix modulates the hallmarks of cancer. EMBO Rep. (2014) 15:1243–53. doi: 10.15252/embr.201439246 25381661 PMC4264927

[34] Qian BZ Pollard JW . Macrophage diversity enhances tumour progression and metastasis. Cell. (2010) 141:39–51. doi: 10.1016/j.cell.2010.03.014 20371344 PMC4994190

[35] Anderson ARA Chaplain MAJ . Continuous and discrete mathematical models of tumour-induced angiogenesis. Bull Math Biol. (1998) 60:857–99. doi: 10.1006/bulm.1998.0042 9739618

[36] Semenza GL . Oxygen sensing, hypoxia-inducible factors, and disease pathophysiology. Annu Rev Pathology: Mech Dis. (2014) 9:47–71. doi: 10.1146/annurev-pathol-012513-104720 23937437

[37] Kalluri R LeBleu VS . The biology, function, and biomedical applications of exosomes. Science. (2020) 367:eaau6977. doi: 10.1126/science.aau6977 32029601 PMC7717626

[38] Buck MD Sowell RT Kaech SM Pearce EL . Metabolic instruction of immunity. Cell. (2017) 169:570–86. doi: 10.1016/j.cell.2017.04.004 28475890 PMC5648021

[39] Orimo A Gupta PB Sgroi DC Arenzana-Seisdedos F Delaunay T Naeem R . Stromal fibroblasts present in invasive human breast carcinomas promote tumour growth and angiogenesis. Cell. (2005) 121:335–48. doi: 10.1016/j.cell.2005.02.034 15882617

[40] Ganjoo S Gupta P Corbali HI Nanez S Riad TS Duong LK . The role of tumour metabolism in modulating T-Cell activity and in optimizing immunotherapy. Front Immunol. (2023) 14:1172931. doi: 10.3389/fimmu.2023.1172931 37180129 PMC10169689

[41] Frades I Foguet C Cascante M Araúzo-Bravo MJ . Genome scale modeling to study the metabolic competition between cells in the tumour microenvironment. Cancers. (2021) 13:4609. doi: 10.3390/cancers13184609 34572839 PMC8470216

[42] Munn DH Mellor AL . IDO in the tumour microenvironment: Inflammation, counter-regulation, and tolerance. Trends Immunol. (2016) 37:193–207. doi: 10.1016/j.it.2016.01.002 26839260 PMC4916957

[43] Rankin EB Giaccia AJ . Hypoxic control of metastasis. Science. (2016) 352:175–80. doi: 10.1126/science.aaf4405 27124451 PMC4898055

[44] Noman MZ Desantis G Janji B Hasmim M Karray S Dessen P . PD-L1 is a novel direct target of HIF-1α, and its blockade under hypoxia enhances MDSC-mediated T cell activation. J Exp Med. (2014) 211:781–90. doi: 10.1084/jem.20131916 24778419 PMC4010891

[45] Domblides C Lartigue L Faustin B . Control of the antitumour immune response by cancer metabolism. Cells. (2019) 8:104. doi: 10.3390/cells8020104 30708988 PMC6406288

[46] Kowal J Tkach M Théry C . Biogenesis and secretion of exosomes. Curr Opin Cell Biol. (2014) 29:116–25. doi: 10.1016/j.ceb.2014.05.004 24959705

[47] Abou Khouzam R Zaarour R Brodaczewska K Azakir B Chouaib S . Hypoxic tumour microenvironment is associated with immune evasion through expression of immune checkpoints and enrichment of suppressive cells. Front Immunol. (2022) 13:828875. doi: 10.3389/fimmu.2022.828875 35211123 PMC8861358

[48] Altrock PM Liu LL Michor F . The mathematics of cancer: Integrating quantitative models. Nat Rev Cancer. (2015) 15:730–45. doi: 10.1038/nrc4029 26597528

[49] Schlesinger M Bendas G . Vascular cell adhesion molecule-1 (VCAM-1)-an increasing insight into its role in tumourigenicity and metastasis. Int J Cancer. (2014) 136:2504–14. doi: 10.1002/ijc.28927 24771582

[50] Boussadia Z Zanetti C Parolini I . Role of microenvironmental acidity and tumour exosomes in cancer immunomodulation. Trans Cancer Res. (2020) 9:5775. doi: 10.21037/tcr.2020.03.69 35117938 PMC8798230

[51] Ariffin AB Forde PF Jahangeer S Soden DM Hinchion J . Releasing pressure in tumours: What do we know so far and where do we go from here? Cancer Res. (2014) 74:2655–62. doi: 10.1158/0008-5472.CAN-13-3696 24778418

[52] Stylianopoulos T Martin JD Chauhan VP Jain SR Diop-Frimpong B Bardeesy N . Causes, consequences, and remedies for growth-induced solid stress in murine and human tumours. Proc Natl Acad Sci. (2012) 109:15101–8. doi: 10.1073/pnas.1213353109 22932871 PMC3458380

[53] Ley K Laudanna C Cybulsky MI Nourshargh S . Getting to the site of inflammation: The leukocyte adhesion cascade updated. Nat Rev Immunol. (2007) 7:678–89. doi: 10.1038/nri2156 17717539

[54] Nourshargh S Alon R . Leukocyte migration into inflamed tissues. Immunity. (2014) 41:694–707. doi: 10.1016/j.immuni.2014.10.008 25517612

[55] Rejniak KA Anderson ARA . Hybrid models of tumour growth. Wiley Interdiscip Reviews: Syst Biol Med. (2011) 3:115–25. doi: 10.1002/wsbm.102 21064037 PMC3057876

[56] Zlotnik A Yoshie O . The chemokine superfamily revisited. Immunity. (2012) 36:705–16. doi: 10.1016/j.immuni.2012.05.008 22633458 PMC3396424

[57] Feig C Jones JO Kraman M Wells RJB Deonarine A Chan DS . Targeting CXCL12 from FAP-expressing carcinoma-associated fibroblasts synergizes with anti–PD-L1 immunotherapy in pancreatic cancer. Proc Natl Acad Sci. (2013) 110:20212–7. doi: 10.1073/pnas.1320318110 24277834 PMC3864274

[58] Mollica Poeta V Massara M Capucetti A Bonecchi R . Chemokines and chemokine receptors: New targets for cancer immunotherapy. Front Immunol. (2019) 10:379. doi: 10.3389/fimmu.2019.00379 30894861 PMC6414456

[59] Kim PS Lee PP Levy D . Modeling regulation mechanisms in the immune system. J Theor Biol. (2007) 246:33–69. doi: 10.1016/j.jtbi.2006.12.012 17270220

[60] Spranger S Dai D Horton B Gajewski TF . Tumour-residing Batf3 dendritic cells are required for effector T cell trafficking and adoptive T cell therapy. Cancer Cell. (2017) 31:711–23. doi: 10.1016/j.ccell.2017.04.003 28486109 PMC5650691

[61] Griffioen AW Damen CA Blijham GH Groenewegen G . Tumour angiogenesis is accompanied by a decreased inflammatory response of tumour-associated endothelium. Blood. (2000) 96:3751–7. doi: 10.1182/blood.V88.2.667.bloodjournal882667 8695814

[62] Weis SM Cheresh DA . Tumour angiogenesis: Molecular pathways and therapeutic targets. Nat Med. (2011) 17:1359–70. doi: 10.1038/nm.2537 22064426

[63] Harjunpää H Llort Asens M Guenther C Fagerholm SC . Cell adhesion molecules and their roles and regulation in the immune and tumour microenvironment. Front Immunol. (2019) 10:1078. doi: 10.3389/fimmu.2019.01078 31231358 PMC6558418

[64] Friedl P Wolf K . Plasticity of cell migration: A multiscale tuning model. J Cell Biol. (2010) 188:11–9. doi: 10.1083/jcb.200909003 19951899 PMC2812848

[65] Galon J Bruni D . Approaches to treat immune hot, altered and cold tumours with combination immunotherapies. Nat Rev Drug Discov. (2019) 18:197–218. doi: 10.1038/s41573-018-0007-y 30610226

[66] Powathil GG Swat M Chaplain MAJ . Systems oncology: Towards patient-specific treatment regimes informed by multiscale mathematical modelling. Semin Cancer Biol. (2015) 30:13–20. doi: 10.1016/j.semcancer.2014.02.003 24607841

[67] Chauhan VP Stylianopoulos T Martin JD Popović Z Chen O Kamoun WS . Normalization of tumour blood vessels improves the delivery of nanomedicines in a size-dependent manner. Nat Nanotechnol. (2012) 7:383–92. doi: 10.1038/nnano.2012.45 22484912 PMC3370066

[68] Shi Y Gilkes DM . HIF-1 and HIF-2 in cancer: structure, regulation, and therapeutic prospects. Cell Mol Life Sci. (2025) 82:44. doi: 10.1007/s00018-024-05537-0 39825916 PMC11741981

[69] Du W Xia X Hu F Yu J . Extracellular matrix remodeling in the tumour immunity. Front Immunol. (2024) 14:1340634. doi: 10.3389/fimmu.2023.1340634 38332915 PMC10850336

[70] Stylianopoulos T . The solid mechanics of cancer and strategies for improved therapy. J Biomech Eng. (2017) 139:021004. doi: 10.1115/1.4034991 27760260 PMC5248974

[71] Xu S Foss F . New nonchemotherapy treatment options for cutaneous T-cell lymphomas. Expert Rev Anticancer Ther. (2021) 21:1017–28. doi: 10.1080/14737140.2021.1882859 33554707

[72] Tokunaga R Zhang W Naseem M Puccini A Berger MD Soni S . CXCL9, CXCL10, CXCL11/CXCR3 axis in cancer. Cancer Treat Rev. (2018) 63:40–7. doi: 10.1016/j.ctrv.2017.11.007 29207310 PMC5801162

[73] Leone P Malerba E Susca N Favoino E Perosa F Brunori G . Endothelial cells in tumour microenvironment: insights and perspectives. Front Immunol. (2024) 15:1367875. doi: 10.3389/fimmu.2024.1367875 38426109 PMC10902062

[74] Kitano H . Biological robustness. Nat Rev Genet. (2004) 5:826–37. doi: 10.1038/nrg1471 15520792

[75] Munn LL Jain RK . Challenges and opportunities for the next generation of computational tumour models. PloS Biol. (2025) 23:e3003269. doi: 10.1371/journal.pbio.3003269 40700394 PMC12286354

[76] Ghaffarizadeh A Heiland R Friedman SH Mumenthaler SM Macklin P . PhysiCell: An open source physics-based cell simulator for 3-D multicellular systems. PloS Comput Biol. (2018) 14:e1005991. doi: 10.1371/journal.pcbi.1005991 29474446 PMC5841829

[77] Kreeger PK Lauffenburger DA . Cancer systems biology: A network modeling perspective. Carcinogenesis. (2010) 31:2–8. doi: 10.1093/carcin/bgp261 19861649 PMC2802670

[78] Breuer K Foroushani AK Laird MR Chen C Sribnaia A Lo R . InnateDB: Systems biology of innate immunity and beyond. Nucleic Acids Res. (2013) 41:D1228–33. doi: 10.1093/nar/gks1147 23180781 PMC3531080

[79] Zhou G Soufan O Ewald J Hancock REW Xia J . NetworkAnalyst 3.0: A visual analytics platform for comprehensive gene expression profiling. Nucleic Acids Res. (2019) 47:W234–41. doi: 10.1093/nar/gkz240 30931480 PMC6602507

[80] Esteva A Kuprel B Novoa RA Ko J Swetter SM Blau HM . Dermatologist-level classification of skin cancer with deep neural networks. Nature. (2017) 542:115–8. doi: 10.1038/nature21056 28117445 PMC8382232

[81] Kipf TN Welling M . Semi-supervised classification with graph convolutional networks. In: International Conference on Learning Representations (ICLR). (2017). Available online at: https://arxiv.org/abs/1609.02907 (Accessed June 20, 2026).

[82] Nyga A Ganguli S Matthews HK Baum B . The role of RAS oncogenes in controlling epithelial mechanics. Trends Cell Biol. (2023) 33:60–9. doi: 10.1016/j.tcb.2022.09.002 36175301 PMC9850021

[83] Weerasinghe HN Mishra S De Silva R . Agent-based modeling approaches for understanding tumour microenvironment dynamics. Math Biosci Eng. (2024) 21:7621–47. doi: 10.3934/mbe.2024335 39696854

[84] Yan DW Wan L Zhang XJ Fang HX Wang SY Zhang MY . New paradigm for rheumatoid arthritis treatment: A review of ‘crosstalk’ mechanism of multi-targeted intervention on cytokine/chemokine networks by Chinese medicine. Chin J Integr Med. (2026) 32:1–11. doi: 10.1007/s11655-025-4232-z 41540163

[85] Ung CY Correia C Zhang Z Caya C Zhu S Billadeau DD . Artificial clinic intelligence (ACI): A generative AI-powered modeling platform to optimize patient cohort enrichment and clinical trial optimization. Cancers. (2025) 17:3543. doi: 10.3390/cancers17213543 41228335 PMC12611047

[86] Azevedo FVPV Ruiz ALTG Rodrigues DS Nakahata DH de Paiva REF de Araujo DR . Navigating the challenges of metallopharmaceutical agents: Strategies and predictive modeling for skin cancer therapy. Pharmaceutics. (2026) 18:145. doi: 10.3390/pharmaceutics18020145 41754887 PMC12943739

[87] Eisenberg MC Jain HV . A confidence building exercise in data and identifiability: Modeling cancer chemotherapy as a case study. J Theor Biol. (2017) 431:63–78. doi: 10.1016/j.jtbi.2017.07.018 28733187 PMC6007023

[88] Preston SP Wilkinson RD Clayton RH Chappell MJ Mirams GR . Think before you fit: parameter identifiability, sensitivity and uncertaintyin systems biology models. Curr Opin Syst Biol. (2025) 52:100563. doi: 10.1016/j.coisb.2025.100563 38826717

[89] Vanin J Hagar A Glazier JA . Who's afraid of synthetic data? Hybrid approaches to deliver medical digital twins. Inf Med Unlocked. (2026) 57:101737. doi: 10.1016/j.imu.2026.101737 41930261 PMC13041779

[90] Moingeon P Chenel M Rousseau C Voisin E Guedj M . Virtual patients, digital twins and causal disease models: Paving the ground for in silico clinical trials. Drug Discov Today. (2023) 28:103605. doi: 10.1016/j.drudis.2023.103605 37146963

[91] Hasin Y Seldin M Lusis A . Multi-omics approaches to disease. Genome Biol. (2017) 18:83. doi: 10.1186/s13059-017-1215-1 28476144 PMC5418815

[92] Racacho KJ Shiau YP Villa R Mahri S Tang M Lin TY . The tumour immune microenvironment: implications for cancer immunotherapy, treatment strategies, and monitoring approaches. Front Immunol. (2025) 16:1621812. doi: 10.3389/fimmu.2025.1621812 41058701 PMC12497833

[93] Armingol E Officer A Harismendy O Lewis NE . Deciphering cell–cell interactions and communication from gene expression. Nat Rev Genet. (2022) 22:71–88. doi: 10.1038/s41576-020-00292-x 33168968 PMC7649713

[94] Wang H Arulraj T Kimko H Popel AS . Generating immunogenomic data-guided virtual patients using a QSP model to predict response of advanced NSCLC to PD-L1 inhibition. NPJ Precis Oncol. (2023) 7:55. doi: 10.1038/s41698-023-00405-9 37291190 PMC10250344

[95] Cheng F Kovács IA Barabási AL . Network-based prediction of drug combinations. Nat Commun. (2022) 10:1197. doi: 10.1038/s41467-019-09186-x 30867426 PMC6416394

[96] Huang Y Kim B Chan C Hahn SM Weissman IL Jiang W . Improving immune–vascular crosstalk for cancer immunotherapy. Nat Rev Immunol. (2018) 18:195–203. doi: 10.1038/nri.2017.145 29332937 PMC5922422

[97] Rafiepoor H Asadi S Ghorbankhanloo A Edalatifard M Abtahi SH Amanpour S . Identification of lung cancer metabolomics profile and molecular interactions using bioinformatic methods. Basic Clin Cancer Res. (2024) 16:119–30. doi: 10.18502/bccr.v16i2.19442

[98] Barberis M . Molecular systems biology of Sic1 in yeast cell cycle regulation through multiscale modeling. In: Advances in Systems Biology. Springer, New York, NY (2012). p. 135–67. doi: 10.1007/978-1-4419-7210-1_7 22161326

[99] Zhang DY Ye F Gao L Liu X Zhao X Che Y . Proteomics, pathway array and signaling network-based medicine in cancer. Cell Div. (2009) 4:20. doi: 10.1186/1747-1028-4-20 19863813 PMC2780394

[100] Sung Y Kim DK Kim JS Kim SJ Kim JH Han JM . Metabolic networks in the tumour microenvironment: roles of amino acid and lipid metabolism pathways in cancer progression and therapy. Exp Mol Med. (2026) 58:1–21. doi: 10.1038/s12276-026-01697-0 41826648 PMC13144319

[101] Kuenzi BM Ideker T . A census of pathway maps in cancer systems biology. Nat Rev Cancer. (2020) 20:233–46. doi: 10.1038/s41568-020-0240-7 32066900 PMC7224610

[102] Chaudhary K Poirion OB Lu L Garmire LX . Deep learning–based multi- omics integration robustly predicts survival in liver cancer. Clin Cancer Res. (2018) 24:1248–59. doi: 10.1158/1078-0432.CCR-17-0853 28982688 PMC6050171

[103] Huang S Chaudhary K Garmire LX . More is better: Recent progress in multi-omics data integration methods. Front Genet. (2023) 8:84. doi: 10.3389/fgene.2017.00084 28670325 PMC5472696

[104] Lotfollahi M Naghipourfar M Luecken MD Khajavi M Büttner M Avsec Ž . Mapping single-cell data to reference atlases by transfer learning. Nat Biotechnol. (2023) 40:121–30. doi: 10.1038/s41587-021-01001-7 34462589 PMC8763644

[105] Jiang J Liu Y Qin J Chen J Wu J Pizzi MP . METI: deep profiling of tumour ecosystems by integrating cell morphology and spatial transcriptomics. Nat Commun. (2024) 15:7312. doi: 10.1038/s41467-024-51708-9 39181865 PMC11344794

[106] Tian J Bai X Quek C . Single-cell informatics for tumour microenvironment and immunotherapy. Int J Mol Sci. (2024) 25:4485. doi: 10.3390/ijms25084485 38674070 PMC11050520

[107] Jiao W Atwal G Polak P Karlic R Cuppen E Danyi A . A deep learning system accurately classifies primary and metastatic cancers using passenger mutation patterns. Nat Commun. (2020) 11:728. doi: 10.1038/s41467-019-13825-8 32024849 PMC7002586

[108] Omar M Dinalankara W Mulder L Coady T Zanettini C Imada EL . Using biological constraints to improve prediction in precision oncology. iScience. (2023) 26:106108. doi: 10.1016/j.isci.2023.106108 36852282 PMC9958363

[109] Myers PJ Lee SH Lazzara MJ . Mechanistic and data-driven models of cell signaling: Tools for fundamental discovery and rational design of therapy. Curr Opin Syst Biol. (2021) 28:100349. doi: 10.1016/j.coisb.2021.05.010. 35935921 PMC9348571

[110] Noordijk B Garcia Gomez ML ten Tusscher KHWJ de Ridder D van Dijk ADJ Smith RW . The rise of scientific machine learning: a perspective on combining mechanistic modelling with machine learning for systems biology. Front Syst Biol. (2024) 4-2024. Available online at: https://www.frontiersin.org/journals/systems-biology/articles/10.3389/fsysb.2024.1407994 (Accessed June 20, 2026). 10.3389/fsysb.2024.1407994 PMC12341957 40809147

[111] Baker RE Peña J-M Jayamohan J Jérusalem A . Mechanistic models versus machine learning: A fight worth fighting for the biological community? Biol Lett. (2018) 14:20170660. doi: 10.1098/rsbl.2017.0660 29769297 PMC6012710

[112] Metzcar J Jutzeler CR Macklin P Köhn-Luque A Brüningk SC . A review of mechanistic learning in mathematical oncology. Front Immunol. (2024) 15-2024. doi: 10.3389/fimmu.2024.1363144 PMC10963621 38533513

[113] Bhattacharya S Andorf S Gomes L Dunn P Schaefer H Pontius J . ImmPort: disseminating data to the public for the future of immunology. Immunol Res. (2014) 58:234–9. doi: 10.1007/s12026-014-8516-1 24791905

[114] Weinstein JN Collisson EA Mills GB Shaw KRM Ozenberger BA Ellrott K . The Cancer Genome Atlas pan-cancer analysis project. Nat Genet. (2013) 45:1113–20. doi: 10.1038/ng.2764 24071849 PMC3919969

[115] Tomczak K Czerwińska P Wiznerowicz M . The Cancer Genome Atlas (TCGA): An immeasurable source of knowledge. Contemp Oncol. (2015) 19:A68–77. doi: 10.5114/wo.2014.47136 25691825 PMC4322527

[116] Colaprico A Silva TC Olsen C Garofano L Cava C Garolini D . TCGAbiolinks: An R/Bioconductor package for integrative analysis of TCGA data. Nucleic Acids Res. (2016) 44:e71. doi: 10.1093/nar/gkv1507 26704973 PMC4856967

[117] Li T Fan J Wang B Traugh N Chen Q Liu JS . TIMER: A web server for comprehensive analysis of tumour-infiltrating immune cells. Cancer Res. (2017) 77:e108–10. doi: 10.1158/0008-5472.CAN-17-0307 29092952 PMC6042652

[118] Li T Fu J Zeng Z Cohen D Li J Chen Q . TIMER2.0 for analysis of tumour-infiltrating immune cells. Nucleic Acids Res. (2020) 48:W509–14. doi: 10.1093/nar/gkaa407 32442275 PMC7319575

[119] Charoentong P Finotello F Angelova M Mayer C Efremova M Rieder D . Pan-cancer immunogenomic analyses reveal genotype– immunophenotype relationships and predictors of response to checkpoint blockade. Cell Rep. (2017) 18:248–62. doi: 10.1016/j.celrep.2016.12.019 28052254

[120] Ru B Wong CN Tong Y Zhong JY Zhong SSW Wu WC . TISIDB: an integrated repository portal for tumour–immune system interactions. Bioinformatics. (2019) 35:4200–2. doi: 10.1093/bioinformatics/btz210 30903160

[121] Tang Z Kang B Li C Chen T Zhang Z . GEPIA2: An enhanced web server for large-scale expression profiling and interactive analysis. Nucleic Acids Res. (2019) 47:W556–60. doi: 10.1093/nar/gkz430 31114875 PMC6602440

[122] Cerami E Gao J Dogrusoz U Gross BE Sumer SO Aksoy BA . The cBio cancer genomics portal: An open platform for exploring multidimensional cancer genomics data. Cancer Discov. (2012) 2:401–4. doi: 10.1158/2159-8290.CD-12-0095 22588877 PMC3956037

[123] Gao J Aksoy BA Dogrusoz U Dresdner G Gross B Sumer SO . Integrative analysis of complex cancer genomics and clinical profiles using the cBioPortal. Sci Signaling. (2013) 6:pl1. doi: 10.1126/scisignal.2004088 23550210 PMC4160307

[124] Regev A Teichmann SA Lander ES Amit I Benoist C Birney E . The human cell atlas. eLife. (2017) 6:e27041. doi: 10.7554/eLife.27041 29206104 PMC5762154

[125] Dong M Thennavan A Urrutia E Li Y Perou CM Zou F . SCDC: bulk gene expression deconvolution by multiple single-cell RNA sequencing references. Briefings Bioinf. (2021) 22:416–27. doi: 10.1093/bib/bbz166 31925417 PMC7820884

[126] White BS de Reyniès A Newman AM Waterfall JJ Lamb A Petitprez F . Community assessment of methods to deconvolve cellular composition from bulk gene expression. Nat Commun. (2024) 15:7362. doi: 10.1038/s41467-024-50618-0 39191725 PMC11350143

[127] Jin H Liu Z . A benchmark for RNA-seq deconvolution analysis under dynamic testing environments. Genome Biol. (2021) 22:102. doi: 10.1186/s13059-021-02290-6 33845875 PMC8042713

[128] Gabriel AAG Racle J Falquet M Jandus C Gfeller D . Robust estimation of cancer and immune cell-type proportions from bulk tumour ATAC-Seq data. Elife. (2024) 13:RP94833. doi: 10.7554/eLife.94833.4 39383060 PMC11464006

[129] Campbell KM Thaker M Medina E Kalbasi A Singh A Ribas A . Spatial profiling reveals association between WNT pathway activation and T-cell exclusion in acquired resistance of synovial sarcoma to NY-ESO-1 transgenic T-cell therapy. J Immunother Cancer. (2022) 10:e004190. doi: 10.1136/jitc-2021-004190 35264439 PMC8915285

[130] Kavran AJ Bai Y Rabe B Kreshock A Fisher A Cheng Y . Spatial genomics reveals cholesterol metabolism as a key factor in colorectal cancer immunotherapy resistance. Front Oncol. (2025) 15:1549237. doi: 10.3389/fonc.2025.1549237 40171265 PMC11959564

[131] Ruiz-Martinez A Gong C Wang H Sové RJ Mi H Kimko H . Simulations of tumour growth and response to immunotherapy by coupling a spatial agent-based model with a whole-patient quantitative systems pharmacology model. PloS Comput Biol. (2022) 18:e1010254. doi: 10.1371/journal.pcbi.1010254 35867773 PMC9348712

[132] Anderson ARA Quaranta V . Integrative mathematical oncology. Nat Rev Cancer. (2008) 8:227–34. doi: 10.1038/nrc2329 18273038

[133] Mariathasan S Turley SJ Nickles D Castiglioni A Yuen K Wang Y . TGF-β attenuates tumour response to PD-L1 blockade by contributing to exclusion of T cells. Nature. (2018) 554:544–8. doi: 10.1038/nature25501 29443960 PMC6028240

[134] Allen RJ Rieger TR Musante CJ . Efficient generation and selection of virtual populations in quantitative systems pharmacology models. CPT: Pharmacometrics Syst Pharmacol. (2016) 5:140–6. doi: 10.1002/psp4.12063 27069777 PMC4809626

[135] Zhu J Paul WE . Heterogeneity and plasticity of T helper cells. Cell Res. (2010) 20:4–12. doi: 10.1038/cr.2009.138 20010916 PMC3494736

[136] Facciabene A Peng X Hagemann IS Balint K Barchetti A Wang L-P . Tumour hypoxia promotes tolerance and angiogenesis via CCL28 and Treg cells. Nature. (2011) 475:226–30. doi: 10.1038/nature10169 21753853

[137] Vivier E Raulet DH Moretta A Caligiuri MA Zitvogel L Lanier LL . Innate or adaptive immunity? The example of natural killer cells. Science. (2011) 331:44–9. doi: 10.1126/science.1198687 21212348 PMC3089969

[138] Romee R Foley B Lenvik T Wang Y Zhang B Ankarlo D . NK cell CD16 surface expression and function is regulated by a disintegrin and metalloprotease-17 (ADAM17). Blood. (2016) 121:3599–608. doi: 10.1182/blood-2012-04-425397 23487023 PMC3643761

[139] Jain RK Martin JD Stylianopoulos T . The role of mechanical forces in tumour growth and therapy. Annu Rev BioMed Eng. (2014) 16:321–46. doi: 10.1146/annurev-bioeng-071813-105259 25014786 PMC4109025

[140] Corrales L McWhirter SM Dubensky TW Gajewski TF . The host STING pathway at the interface of cancer and immunity. J Clin Invest. (2016) 126:2404–11. doi: 10.1172/JCI86892 27367184 PMC4922692

[141] Neal JT Li X Zhu J Giangarra V Grzeskowiak CL Ju J . Organoid modeling of the tumour immune microenvironment. Cell. (2018) 175:1972–1988.e16. doi: 10.1016/j.cell.2018.11.021 30550791 PMC6656687

[142] Biswas SK Mantovani A . Macrophage plasticity and interaction with lymphocyte subsets: Cancer as a paradigm. Nat Immunol. (2010) 11:889–96. doi: 10.1038/ni.1937 20856220

[143] Ries CH Cannarile MA Hoves S Benz J Wartha K Runza V . Targeting tumour-associated macrophages with anti-CSF-1R antibody reveals a strategy for cancer therapy. Cancer Cell. (2014) 25:846–59. doi: 10.1016/j.ccr.2014.05.016 24898549

[144] Colegio OR Chu NQ Szabo AL Chu T Rhebergen AM Jairam V . Functional polarization of tumour-associated macrophages by tumour-derived lactic acid. Nature. (2014) 513:559–63. doi: 10.1038/nature13490 25043024 PMC4301845

[145] Veglia F Perego M Gabrilovich D . Myeloid-derived suppressor cells coming of age. Nat Immunol. (2018) 19:108–19. doi: 10.1038/s41590-017-0022-x 29348500 PMC5854158

[146] Highfill SL Cui Y Giles AJ Smith JP Zhang H Morse E . Disruption of CXCR2-mediated MDSC tumour trafficking enhances anti–PD1 efficacy. Sci Transl Med. (2014) 6:237ra67. doi: 10.1126/scitranslmed.3007974 24848257 PMC6980372

[147] Sharma P Hu-Lieskovan S Wargo JA Ribas A . Primary, adaptive, and acquired resistance to cancer immunotherapy. Cell. (2017) 168:707–23. doi: 10.1016/j.cell.2017.01.017 28187290 PMC5391692

[148] Ayers M Lunceford J Nebozhyn M Murphy E Loboda A Kaufman DR . IFN- γ–related mRNA profile predicts clinical response to PD-1 blockade. J Clin Invest. (2017) 127:2930–40. doi: 10.1172/JCI91190 28650338 PMC5531419

[149] Samstein RM Lee CH Shoushtari AN Hellmann MD Shen R Janjigian YY . Tumour mutational load predicts survival after immunotherapy across multiple cancer types. Nat Genet. (2019) 51:202–6. doi: 10.1038/s41588-018-0312-8 30643254 PMC6365097

[150] Lan Y Zhang D Xu C Hance KW Marelli B Qi J . Enhanced preclinical antitumour activity of M7824, a bifunctional fusion protein targeting PD-L1 and TGF-β. Sci Transl Med. (2018) 10:eaan5488. doi: 10.1126/scitranslmed.aan5488 29343622

[151] Juneja VR McGuire KA Manguso RT LaFleur MW Collins N Haining WN . PD-L1 on tumour cells is sufficient for immune evasion in immunogenic tumours. J Exp Med. (2017) 214:895–904. doi: 10.1084/jem.20160801 28302645 PMC5379970

[152] Cristescu R Mogg R Ayers M Albright A Murphy E Yearley J . Pan-tumour genomic biomarkers for PD-1 checkpoint blockade-based immunotherapy. Science. (2018) 362:eaar3593. doi: 10.1126/science.aar3593 30309915 PMC6718162

[153] Tawbi HA SChadendorf D Lipson EJ Ascierto PA Matamala L Castillo Gutiérrez E . Relatlimab and nivolumab versus nivolumab in untreated advanced melanoma. N Engl J Med. (2022) 386:24–34. doi: 10.1056/NEJMoa2109970 34986285 PMC9844513

[154] Esensten JH Helou YA Chopra G Weiss A Bluestone JA . CD28 costimulation: From mechanism to therapy. Immunity. (2016) 44:973–88. doi: 10.1016/j.immuni.2016.04.020 27192564 PMC4932896

[155] Wei SC Duffy CR Allison JP . Fundamental mechanisms of immune checkpoint blockade therapy. Cancer Discov. (2018) 8:1069–86. doi: 10.1158/2159-8290.CD-18-0367 30115704

[156] Larkin J Chiarion-Sileni V Gonzalez R Grob JJ Cowey CL Lao CD . Combined nivolumab and ipilimumab or monotherapy in untreated melanoma. N Engl J Med. (2015) 373:23–34. doi: 10.1056/NEJMoa1504030 26027431 PMC5698905

[157] Riaz N Havel JJ Makarov V Desrichard A Urba WJ Sims JS . Tumour and microenvironment evolution during immunotherapy with nivolumab. Cell. (2017) 171:934–949.e16. doi: 10.1016/j.cell.2017.09.028 29033130 PMC5685550

[158] Callahan MK Wolchok JD Allison JP . Anti–CTLA-4 antibody therapy: Immune monitoring during clinical development of a novel immunotherapy. Semin Oncol. (2010) 37:473–84. doi: 10.1053/j.seminoncol.2010.09.001 21074063 PMC3008567

[159] Anderson AC Joller N Kuchroo VK . Lag-3, Tim-3, and TIGIT: Co- inhibitory receptors with specialized functions in immune regulation. Immunity. (2016) 44:989–1004. doi: 10.1016/j.immuni.2016.05.001 27192565 PMC4942846

[160] Johnston RJ Comps-Agrar L Hackney J Yu X Huseni M Yang Y . The immunoreceptor TIGIT regulates antitumour and antiviral CD8+ T cell effector function. Cancer Cell. (2014) 26:923–37. doi: 10.1016/j.ccell.2014.10.018 25465800

[161] Chen H Xu Z Varner J . Targeting myeloid cells to improve cancer immune therapy. Front Immunol. (2025) 16:1623436. doi: 10.3389/fimmu.2025.1623436 40821795 PMC12350267

[162] Buchbinder EI Desai A . CTLA-4 and PD-1 pathways: Similarities, differences, and implications of their inhibition. Am J Clin Oncol. (2016) 39:98–106. doi: 10.1097/COC.0000000000000239 26558876 PMC4892769

[163] Yarchoan M Hopkins A Jaffee EM . Tumour mutational burden and response rate to PD-1 inhibition. N Engl J Med. (2017) 377:2500–1. doi: 10.1056/NEJMc1713444 29262275 PMC6549688

[164] Dries R Zhu Q Dong R Eng CHL Li H Liu K . Giotto: A toolbox for integrative analysis and visualization of spatial expression data. Genome Biol. (2021) 22:78. doi: 10.1186/s13059-021-02286-2 33685491 PMC7938609

[165] Reel PS Reel S Pearson E Trucco E Jefferson E . Using machine learning approaches for multi-omics data analysis: A review. Biotechnol Adv. (2021) 49:107739. doi: 10.1016/j.bioteChadv.2021.107739 33794304

[166] Langfelder P Horvath S . WGCNA: An R package for weighted correlation network analysis. BMC Bioinf. (2008) 9:559. doi: 10.1186/1471-2105-9-559 19114008 PMC2631488

[167] Mayakonda A Lin D-C Assenov Y Plass C Koeffler HP . Maftools: Efficient and comprehensive analysis of somatic variants in cancer. Genome Res. (2018) 28:1747–56. doi: 10.1101/gr.239244.118 30341162 PMC6211645

[168] Racle J de Jonge K Baumgaertner P Speiser DE Gfeller D . Simultaneous enumeration of cancer and immune cell types from bulk tumour gene expression data. eLife. (2017) 6:e26476. doi: 10.7554/eLife.26476 29130882 PMC5718706

[169] Argelaguet R Velten B Arnol D Dietrich S Zenz T Marioni JC . Multi-omics factor analysis—a framework for unsupervised integration of multi-omics data sets. Mol Syst Biol. (2018) 14:e8124. doi: 10.15252/msb.20178124 29925568 PMC6010767

[170] Subramanian I Verma S Kumar S Jere A Anamika K . Multi-omics data integration, interpretation, and its application. Bioinf Biol Insights. (2020) 14:1177932219899051. doi: 10.1177/1177932219899051 32076369 PMC7003173

[171] Failmezger H Zwing N Tresch A Korski K Schmich F . Computational tumour infiltration phenotypes enable the spatial and genomic analysis of immune infiltration in colorectal cancer. Front Oncol. (2021) 11:552331. doi: 10.3389/fonc.2021.552331 33791196 PMC8006941

[172] Desbois M Udyavar AR Ryner L Kozlowski C Guan Y Dürrbaum M . Integrated digital pathology and transcriptome analysis identifies molecular mediators of T-cell exclusion in ovarian cancer. Nat Commun. (2020) 11:5583. doi: 10.1038/s41467-020-19408-2 33149148 PMC7642433

[173] Hänzelmann S Castelo R Guinney J . GSVA: Gene set variation analysis for microarray and RNA-seq data. BMC Bioinf. (2013) 14:7. doi: 10.1186/1471-2105-14-7 23323831 PMC3618321

[174] Jerby-Arnon L Shah P Cuoco MS Rodman C Su M-J Melms JC . A cancer cell program promotes T cell exclusion and resistance to checkpoint blockade. Cell. (2018) 175:984–997.e24. doi: 10.1016/j.cell.2018.09.006 30388455 PMC6410377

[175] Argelaguet R Arnol D Bredikhin D Deloro Y Velten B Marioni JC . MOFA+: A statistical framework for comprehensive integration of multi-modal single-cell data. Genome Biol. (2020) 21:111. doi: 10.1186/s13059-020-02015-1 32393329 PMC7212577

[176] Jackson HW Fischer JR Zanotelli VRT Ali HR Mechera R Soysal SD . The single-cell pathology landscape of breast cancer. Nature. (2020) 578:615–20. doi: 10.1038/s41586-019-1876-x 31959985

[177] Moscardó García M Aalto A Montanari AN Skupin A Gonçalves J . Multi-omic network inference from time-series data. NPJ Syst Biol Appl. (2025) 11:114. doi: 10.1038/s41540-025-00591-1 41087386 PMC12521560

[178] Berglund E Maaskola J Schultz N Friedrich S Marklund M Bergenstråhle J . Spatial maps of prostate cancer transcriptomes reveal an unexplored landscape of heterogeneity. Nat Commun. (2018) 9:2419. doi: 10.1038/s41467-018-04724-5 29925878 PMC6010471

[179] Ståhl PL Salmén F Vickovic S Lundmark A Navarro JF Magnusson J . Visualization and analysis of gene expression in tissue sections by spatial transcriptomics. Science. (2016) 353:78–82. doi: 10.1126/science.aaf2403 27365449

[180] Efremova M Vento-Tormo M Teichmann SA Vento-Tormo R . CellPhoneDB: Inferring cell–cell communication from combined expression of multi- subunit receptor–ligand complexes. Nat Protoc. (2020) 15:1484–506. doi: 10.1038/s41596-020-0292-x 32103204

[181] Jin S Guerrero-Juarez CF Zhang L Chang I Ramos R Kuan C-H . Inference and analysis of cell–cell communication using CellChat. Nat Commun. (2021) 12:1088. doi: 10.1038/s41467-021-21246-9 33597522 PMC7889871

[182] Satija R Farrell JA Gennert D Schier AF Regev A . Spatial reconstruction of single-cell gene expression data. Nat Biotechnol. (2015) 33:495–502. doi: 10.1038/nbt.3192 25867923 PMC4430369

[183] Yu Y Mai Y Zheng Y Shi L . Assessing and mitigating batch effects in large-scale omics studies. Genome Biol. (2024) 25:254. doi: 10.1186/s13059-024-03401-9 39363244 PMC11447944

[184] Lê Cao KA Abadi AJ Davis-Marcisak EF Hsu L Arora A Coullomb A . Community-wide hackathons to identify central themes in single-cell multi-omics. Genome Biol. (2021) 22:220. doi: 10.1186/s13059-021-02433-9 34353350 PMC8340473

[185] Topol EJ . High-performance medicine: The convergence of human and artificial intelligence. Nat Med. (2019) 25:44–56. doi: 10.1038/s41591-018-0300-7 30617339

[186] Cabrita R Lauss M Sanna A Donia M Skaarup Larsen M Mitra S . Tertiary lymphoid structures improve immunotherapy and survival in melanoma. Nature. (2020) 577:561–5. doi: 10.1038/s41586-019-1914-8 31942071

[187] Hicks SC Townes FW Teng M Irizarry RA . Missing data and technical variability in single-cell RNA-sequencing experiments. Biostatistics. (2018) 19:562–78. doi: 10.1093/biostatistics/kxx053 29121214 PMC6215955

[188] Lähnemann D Köster J Szczurek E McCarthy DJ Hicks SC Robinson MD . Eleven grand challenges in single-cell data science. Genome Biol. (2020) 21:31. doi: 10.1186/s13059-020-1926-6 32033589 PMC7007675

[189] Stuart T Butler A Hoffman P Hafemeister C Papalexi E Mauck WM . Comprehensive integration of single-cell data. Cell. (2019) 177:1888–1902.e21. doi: 10.1016/j.cell.2019.05.031 31178118 PMC6687398

[190] Trapnell C Cacchiarelli D Grimsby J Pokharel P Li S Morse M . The dynamics and regulators of cell fate decisions revealed by pseudotemporal ordering of single cells. Nat Biotechnol. (2014) 32:381–6. doi: 10.1038/nbt.2859 24658644 PMC4122333

[191] Karczewski KJ Snyder MP . Integrative omics for health and disease. Nat Rev Genet. (2018) 19:299–310. doi: 10.1038/nrg.2018.4 29479082 PMC5990367

[192] Creswell A White T Dumoulin V Arulkumaran K Sengupta B Bharath AA . Generative adversarial networks: An overview. IEEE Signal Process Mag. (2018) 35:53–65. doi: 10.1109/MSP.2017.2765202 25079929

[193] Doshi-Velez F Kim B . Towards a rigorous science of interpretable machine learning. arXiv. (2017). Available online at: https://arxiv.org/abs/1702.08608. preprint arXiv:1702.08608.

[194] Rieke N Hancox J Li W Milletari F Roth HR Albarqouni S . The future of digital health with federated learning. NPJ Digital Med. (2020) 3:119. doi: 10.1038/s41746-020-00323-1 33015372 PMC7490367

[195] Bruynseels K Santoni de Sio F van den Hoven J . Digital twins in health care: Ethical implications of an emerging engineering paradigm. Front Genet. (2018) 9:31. doi: 10.3389/fgene.2018.00031 29487613 PMC5816748

[196] Kaissis G Makowski M Rückert D Braren RF . Secure, privacy-preserving and federated machine learning in medical imaging. Nat Mach Intell. (2020) 2:305–11. doi: 10.1038/s42256-020-0186-1 37880705

[197] Lin Y Huang Z Lin Z Lin Y Song J Luo L . TiRank prioritizes phenotypic niches in tumor microenvironment for clinical biomarker discovery. Genome Med. (2026) 18:23. doi: 10.1186/s13073-026-01604-2 41689080 PMC12910759

[198] La Manno G Soldatov R Zeisel A Braun E Hochgerner H Petukhov V . RNA velocity of single cells. Nature. (2018) 560:494–8. doi: 10.1038/s41586-018-0414-6 30089906 PMC6130801

[199] Ding T Wagner SJ Song AH Chen RJ Lu MY Zhang A . A multimodal whole-slide foundation model for pathology. Nat Med. (2025) 31:1–13. doi: 10.1038/s41591-025-03982-3 41193692 PMC12618242

[200] Liu T Huang T Ding T Wu H Humphrey P Perincheri S . Leveraging multi-modal foundation models for analysing spatial multi-omic and histopathology data. Nat BioMed Eng. (2026) 10:1–18. doi: 10.1038/s41551-025-01602-6 41644824

[201] Larson CR Mandloi A Acharyya S Carstens JL . The tumour microenvironment across four dimensions: assessing space and time in cancer biology. Front Immunol. (2025) 16:1554114. doi: 10.3389/fimmu.2025.1554114 40625731 PMC12230089

[202] Collins FS Varmus H . A new initiative on precision medicine. N Engl J Med. (2015) 372:793–5. doi: 10.1056/NEJMp1500523 25635347 PMC5101938

[203] Swanson KR Rostomily RC Alvord EC . A mathematical modelling tool for predicting survival of individual patients following resection of glioblastoma. Br J Cancer. (2008) 98:1–4. doi: 10.1038/sj.bjc.6604125 18059395 PMC2359692

[204] Kirschner D Panetta JC . Modeling immunotherapy of the tumour–immune interaction. J Math Biol. (1998) 37:235–52. doi: 10.1007/s002850050127 9785481

[205] Sharma P Allison JP . The future of immune checkpoint therapy. Science. (2015) 348:56–61. doi: 10.1126/science.aaa8172 25838373

[206] Fridman WH Zitvogel L Sautès‐Fridman C Kroemer G . The immune contexture in cancer prognosis and treatment. Nat Rev Clin Oncol. (2017) 14:717–34. doi: 10.1038/nrclinonc.2017.101 28741618

[207] Byrne HM . Dissecting cancer through mathematics: From the cell to the animal model. Nat Rev Cancer. (2010) 10:221–30. doi: 10.1038/nrc2808 20179714

[208] Viceconti M Henney A Morley-Fletcher E . In silico clinical trials: How computer simulation will transform the biomedical industry. Trends Biotechnol. (2016) 34:998–1007. doi: 10.1016/j.tibtech.2016.05.012 27325423

